# Molecular mechanisms and pathobiology of oncogenic fusion transcripts in epithelial tumors

**DOI:** 10.18632/oncotarget.26777

**Published:** 2019-03-12

**Authors:** Musaffe Tuna, Christopher I. Amos, Gordon B. Mills

**Affiliations:** ^1^ Department of Epidemiology, The University of Texas MD Anderson Cancer Center, Houston, TX, USA; ^2^ Department of Medicine, Baylor College of Medicine, Houston, TX, USA; ^3^ Institute for Clinical and Translational Research, Baylor College of Medicine, Houston, TX, USA; ^4^ Department of Systems Biology, The University of Texas MD Anderson Cancer Center, Houston, TX, USA; ^5^ Department of Cell, Developmental and Cancer Biology, School of Medicine, Oregon Health Science University, Portland, OR, USA; ^6^ Precision Oncology, Knight Cancer Institute, Portland, OR, USA

**Keywords:** fusion genes, fusion transcripts, epithelial tumors, mechanisms, pathobiology

## Abstract

Recurrent fusion transcripts, which are one of the characteristic hallmarks of cancer, arise either from chromosomal rearrangements or from transcriptional errors in splicing. DNA rearrangements include intrachromosomal or interchromosomal translocation, tandem duplication, deletion, inversion, or result from chromothripsis, which causes complex rearrangements. In addition, fusion proteins can be created through transcriptional read-through. Fusion genes can be transcribed to fusion transcripts and translated to chimeric proteins, with many having demonstrated transforming activities through multiple mechanisms in cells. Fusion proteins represent novel therapeutic targets and diagnostic biomarkers of diagnosis, disease status, or progression. This review focuses on the mechanisms underlying the formation of oncogenic fusion genes and transcripts and their impact on the pathobiology of epithelial tumors.

## INTRODUCTION

Three decades’ worth of accumulating data have shown that chromosomal rearrangements, including fusion genes, are frequent events and can act as genetic drivers of hematologic malignancies and mesenchymal tumors [1–3]. However, the occurrence and the biological and clinical effects of gene fusions in epithelial tumors have been less well recognized. The first fusion gene found in epithelial tumors (*RET-CCDC6*) was discovered in papillary thyroid carcinoma in the early 1990s [4, 5]. Other fusion gene discoveries soon followed in epithelial tumors and other malignancies [6]. Since then, even though the finding of novel fusion genes in epithelial tumors has increased tremendously, they remained poorly described until the last decade. Technical limits once precluded their characterization, but recent rapid advances in genome-sequencing technologies, analytical tools, and application of these technologies have propelled the discovery of fusion genes in epithelial cancers. Such discoveries have revealed thousands of fusion transcripts in hematologic malignancies and mesenchymal tumors as well as in epithelial tumors. However, only a small fraction has been assessed for transforming ability and mechanistic alterations, leaving a large fraction for functional characterization. Therefore, functional characterization of fusion genes needs to be performed to identify those that are ‘driver’ mutations rather than ‘passenger’ mutations and importantly to ensure that the proposed fusion genes are indeed expressed and functional in different tumor lineages. Furthermore, an improved understanding of the mechanisms underlying the formation of fusion transcripts and their effect on cell function will be crucial to translate the rapidly emerging ability to identify fusion transcripts to clinical application. The protein products of fusion genes can serve as therapeutic targets or as biomarkers for diagnosis or disease progression. Here, we focus on mechanisms that lead to the development of fusion genes and alternate transcript formation and the subsequent pathobiology of recurrent gene fusions.

## FUSION GENES IN EPITHELIAL TUMORS

Accumulated data in human cancers from recent efforts by The Cancer Genome Atlas (TCGA), International Collaboration for Clinical Genomics (ICCG), International Cancer Genome Consortium (ICGC), and individually generated studies provide evidence that fusion genes/transcripts are much more common in epithelial tumors than previously thought. Indeed, the latest discoveries have demonstrated that fusion genes can be drivers in epithelial tumors in the same way as in hematologic malignancies and mesenchymal tumors. Initially, the genes involved in fusion formation were mainly classified into two classes: 1) transcription factors (Supplementary Figure 1B) or associated cofactors (e.g., *RARA, MYC*) and 2) receptor and non-receptor tyrosine kinases (e.g., *ABL1, ALK, FGFR, NTRK*) (Supplementary Figure 1A). In the last decade, genomic analyses dramatically changed this view so that a broader spectrum of genes was recognized as being involved in the formation of fusion genes and consequently in carcinogenic transformation. As described herein, genes involved in fusion formation that are implicated in transformation, progression, or resistance to therapy extend beyond tyrosine kinases and transcription factors or associated cofactors and include genes that mediate nucleo-cytoplasmic transport of protein and RNA (e.g., nucleoporin), a lysine-specific methyltransferase (e.g., *MLL*), and genes that are involved in metabolic pathways (e.g., *PLAG2G4B*), signal transduction (e.g., R-spondin in Wnt signaling), DNA repair (e.g., *RAD51C*), chromosome segregation (e.g., *TACC*), tumor suppressor genes (e.g. *TP53, PTEN, CBFB*), and oncogenes (e.g. *GNAS, ERBB2*). However, the role of most fusion genes in tumorigenesis remains unknown. While most fusion genes are tumor specific (and indeed many are private and found only in single tumors) [7], some fusion genes are involved in the tumorigenesis of multiple epithelial cancers as well as hematologic malignancies [8] and mesenchymal tumors.

## FORMATION OF FUSION GENES AND BIOLOGICAL IMPACT OF ONCOGENESIS

**Figure 1:**
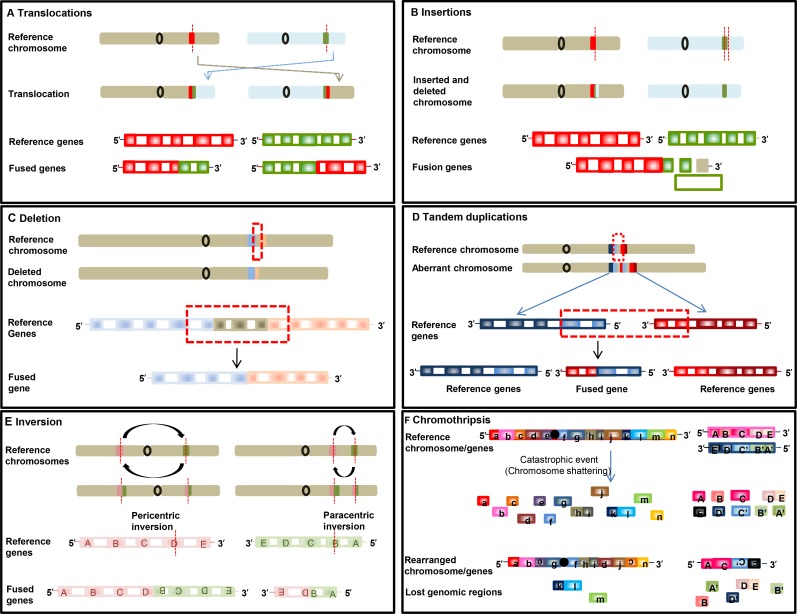
Schematic illustrations of formation of fusion genes Formation of fusion gene via translocation (**A**), insertion (**B**), deletion (**C**), tandem duplication (**D**), inversion (**E**), chromothripsis (**F**).

**Figure 2:**
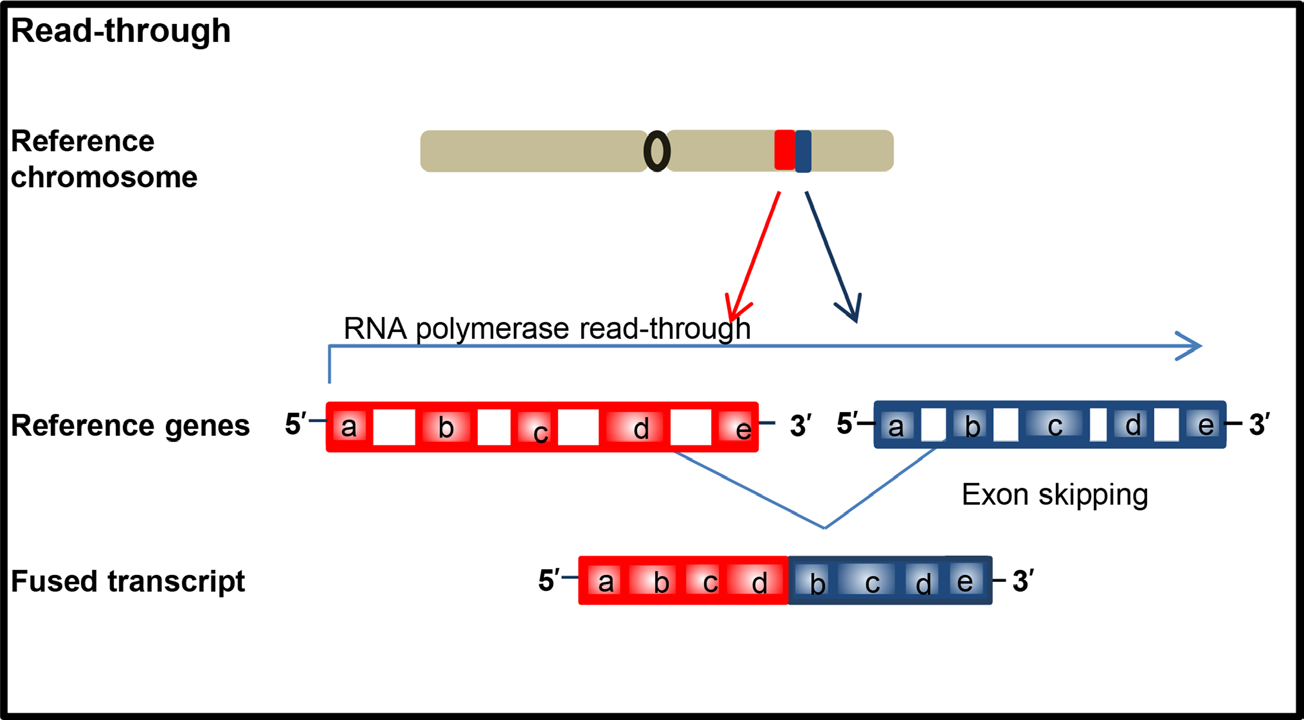
Schematic illustration of formation of chimeric transcript

The first fusion gene discovered in human cancer was the *BCR-ABL* gene, described in 1960. The breakpoint cluster region (*BCR)-*c-abl oncogene, non-receptor tyrosine kinase *(ABL)* fusion gene is the result of a reciprocal translocation between the q arms of chromosomes 9 and 22 (i.e., an interchromosomal translocation; Figure 1A) and occurs in more than 96% of patients with chronic myelogenous leukemia (CML) [2]. Soon after, the discovery of fusion genes involving *MYC* and promyelocytic leukemia (*PML*) followed. Fusions caused by interchromosomal translocation are thought to account for only a fraction of fusion genes. Recent technological advances have revealed that, in addition to being caused by interchromosomal translocations, fusion transcripts also arise by intrachromosomal translocation, insertion, deletion, tandem duplication, inversion, chromothripsis, and aberrant splicing (read-through) (Figure 2).

### Pathogenesis of fusion gene formation

As aforementioned, oncogenic fusion genes or transcripts can arise through multiple different types of rearrangement (Figures 1 and 2), which, with two exceptions, create tail-to-tail and head-to-head fusions. Fusion genes can occur through alterations at the DNA level by six different rearrangements (Figure 1). Furthermore, fusion transcripts can form through read-through during RNA transcription without structural chromosomal changes (Figure 2).

The *first* type of rearrangement is reciprocal translocation. It can be balanced or unbalanced interchromosomal translocation. Balanced translocation is caused by an exchange of DNA sequences without missing or extra genetic information between two different chromosomes (Figure 1A). Unbalanced translocation, in which the exchange of sequences is unequal, results in missing or extra genetic information. As shown in Supplementary Table 1, fusion of solute carrier family 34, member 2 (*SLC34A2*) with the gene encoding c-ros oncogene 1 (*ROS1*) in non-small cell lung cancer (NSCLC) [9, 10] is an example of reciprocal translocation.

The *second* type of rearrangement is insertion. Insertions are due to movement of a DNA fragment from one region into another in the same chromosome (intrachromosomal) or from one chromosome into other (interchromosomal), the latter also known as nonreciprocal translocation (Figure 1B).

The *third* type of rearrangement is the formation of fusion genes by juxtaposition of two genes through deletion of regions between two genes that transcribe in the same direction (Figure 1C). Fusion genes such as *ATG7-RAF1* in pancreatic cancer (Supplementary Table 2) and *EIF3E-RSPO2* in colon cancer are examples of fusion resulting from deletion [7].

The *fourth* type of rearrangement is tandem duplication. In this scenario, a genomic region is duplicated, resulting in a fusion with a gene in the original region. Fibroblast growth factor receptor 3 (*FGFR3)-*transforming, acidic coiled-coil containing protein 3 *(TACC3)* in glioblastoma [11] and chromosome 2 open reading frame 14 (*C2orf44)-ALK* fusions in colorectal cancer [12] are examples of tandem duplications (Figure 1D).

The *fifth* type of rearrangement is inversion, in which chromosomal segments flip with (pericentric) or without (paracentric) relationship with the centromere (Figure 1E); examples include kinesin family member 5B (*KIF5B)-RET* in lung adenocarcinoma [12] and echinoderm microtubule-associated protein-like 4 (*EML4)-ALK* in non–small cell lung cancer [13].

The *sixth* type of rearrangement is chromothripsis, in which fusion arises when one chromosome or chromosome region or a few chromosomes shatter into many fragments, and fragments reassemble inaccurately (Figure 1F). Examples of this characteristic event are *PVT1-MYC* and *PVT1-NDRG1* fusions in medulloblastoma [14], and *NDUFAF2-MAST4* in prostate cancer cell lines [15].

Aberrant fusion transcript formation can occur due to read-through transcripts, which is different from the other six rearrangements due to occurring at the RNA level. Importantly, this is the only type of fusion transcript that does not involve rearrangement of genomic material. Chimeric transcripts arise when an RNA polymerase does not properly terminate transcription at the end of a gene and continues transcribing until the end of the following gene due to aberrant splicing (Figure 2). Chimeric protein of FGFR3-BAI associated protein 2 like 1 (BAIAP2L1) in bladder carcinoma is example of this rearrangement [16]. This type of rearrangement is not restricted to cancer as genes such as MDS1 and EVI1 can create the read through MECOM in normal or malignant cells.

Interestingly, formation of the same fusion can occur through different rearrangements. An example of this scenario is *TMPRSS2-EGR* fusion (Supplementary Table 3), which can arise through inversion of chromosome 21q22 in some cases and by interstitial deletion of chromosome 21q22 in other cases [17]. When fusion genes are caused by deletion or tandem duplication, both genes are likely located on the same direction and strand of a chromosome, whereas in fusion genes caused by inversion, the two genes are more likely to be found on opposite chromosome strands. Notably, fusion genes can be complex by involving multiple genes. Fusion genes that express a fusion protein can gain functional properties, or alternatively there can be increased amounts or activity of a single component of the fusion gene. Both mechanisms can lead to cell transformation.

### Heterogeneity in fusion genes

Fusion genes can demonstrate marked heterogeneity based on different partners, variable breakpoints, co-mutations, and tissue specific effects. For example ALK, ROS1, and BRAF can have multiple fusion partners and different breakpoints (Supplementary Figure 1) that can alter functional and therapeutic consequences of the fusion genes.

### Processes underlying the formation of fusion genes

Repair of double-strand breaks caused by defects in DNA damage repair or bridge-breakage-fusion events [18] by canonical non-homologous end joining (NHEJ) [19], alt-NHEJ [20, 21], break induced replication repair or synthesis induced end joining [21] can alter the structure of the breaks. Further, interphase gene proximity (spatial proximity) is tissue specific and can facilitate generation of fusion genes. For example *PML* and *RARa* are found in close spatial proximity in hematopoietic cells resulting in frequent fusion in hematopoietic but not other cells [22]. Similar spatial proximity during interphase has been proposed to mediate *RET* and *H4* fusion in papillary thyroid carcinoma [23], and *TMPRSS2* and *ERG* in prostate cancer [24]. Thus understanding the processes leading to the formation of fusion genes could alter the functional and therapeutic relevance of fusion genes.

### Pathobiology of oncogenic fusion genes

Fusion genes can influence biology through six mechanisms. *A first mechanism* is overexpression of an oncogene through promoter exchange or through linking of an open reading frame to transcriptional control elements (e.g., *TMPRSS2* to *ETS*, *IgH* to *MYC*, *IgH* to *BCL2*) that are active in the target tissue. As seen in prostate cancer, the forced expression of normal proteins (i.e. 3′ gene, ETS family of transcription factors) through an androgen-responsive promoter (TMPRSS2, a transmembrane serine protease) or by fusion to ubiquitous promoters results in overexpression of a wild-type protein [17, 25–27]. Where the major consequence of a fusion is overexpression of a wild-type protein. *TMPRSS2-ERG* is the most common fusion in prostate adenocarcinomas (50%) and in precursor high-grade prostatic intraepithelial neoplasia (approximately 20%) [26, 28], and promotes metastasis to bone [29]. *TMPRSS2* has been shown to fuse to 20 different partners in prostate adenocarcinomas (Supplementary Table 3) [26, 28]. ETS family members can also be fused with other 5′ partners (e.g., *SLC45A3,* kallikrein 2 (*KLK2*), and calcium-activated nucleotidase 1 (*CANT1*)) [30, 31].

*A second mechanism* affects biology by causing truncations, which may result in loss of negative regulatory microRNA binding sites. *MYB-*nuclear factor I/B (*NFIB*) fusion results in loss of miR-15a/16 and miR-150 binding sites, thereby resulting in overexpression of MYB protein [32]. This fusion occurs in adenoid cystic carcinoma of the breast (67%) and salivary gland (28%) as well as in benign sporadic, dermal cylindromas (60%) [33]. Similar mechanisms occur in other fusion genes such as high mobility group AT-Hook2 (HMGA2)-NFIB, in which loss of negatively regulating let-7 miRNA–binding sequences in the 3′ UTR of HMGA2 enhances oncogenic transformation [34]. This fusion is recurrent in benign pleomorphic salivary gland adenomas [35]. The *FGFR3-TACC3* fusion also loses a negatively regulatory binding site of miR-99a, which causes overexpression of the protein and contributes to aneuploidy in glioblastoma cells [11]. As a further example, *TFEB*–*CADM2* fusions, which occur in type 2 papillary renal cell carcinoma, also lose several miRNA-binding sites, [36].

*The third mechanism* influences cell function by destroying the intrinsic control mechanism through introducing dimerization or oligomerization domain(s) of fusion partner genes. A dimerization or oligomerization domain of partner proteins leads to constitutive activation of the chimeric protein itself as well as downstream signaling pathways. The best example of this is the first identified fusions of *BCR-ABL1* in CML. The ABL1 kinase is constitutively activated by BCR-ABL1 oligomerization by the coiled-coil domain in BCR and is necessary for the transforming activity of ABL [37]. Transmembrane TK are activated by ligand binding to their extracellular domains, which leads to receptor dimerization and transphosphorylation of tyrosine residues located in the kinase activation loop or in binding sites for linker molecules, thus activating downstream signaling pathways. Fusion proteins frequently co-opt the normal activation mechanism of tyrosine kinases through dimerization domains in partner proteins. Notably, most if not all transmembrane tyrosine-kinase fusion gene (e.g., *ALK*, *FGFR*, *NTRK*, *RET*, and *ROS1*) partners contain dimerization or oligomerization domains; either coiled-coil or SAM, LisH, BAR, and SPFH [16, 38–41]. The TK frequently fuse with multiple partner genes. For instance, anaplastic lymphoma kinase gene (*ALK*) fuses with *CLTC, TPM3, TFG, ATIC, KIFB, C2orf44*, and *EML4* (Supplementary Figure 1C), which all contain dimerization domains, rendering ALK constitutively active and resulting in activation of downstream signaling pathways. Fusions that lead to ALK activation can promote cell transformation, proliferation, aberrant development, and clonal expansion. Activation of wild-type ALK induces downstream signaling pathways including phosphatidylinositol 3-kinase–AKT, mitogen-activated protein kinase kinase (MEK)–extracellular signal-regulated kinase (ERK), and STAT [8]. However, the EML4-ALK chimeric protein activates AKT and ERK pathways in EGFR mutation–positive NSCLC [42], while NPM-ALK fusion promotes ERK and STAT3 pathways in anaplastic large cell lymphoma [43]. Thus downstream signaling pathways and roles of chimeric proteins can vary in the context of different cancer subtypes. ALK fusions are frequently observed in glioblastoma, non-small lung cancer, and papillary thyroid cancers (Supplementary Table 1).

FGFR TK family (FGFR1, FGFR2, FGFR3, and FGFR4) members encode transmembrane proteins that contain immunoglobulin-like and kinase domains (Figure 2) that play diverse roles in controlling cell proliferation, cell differentiation, angiogenesis, and tumor development. The most common fusion partners of *FGFR1* or *FGFR3* are the transforming acidic coiled-coil (TACC)-coding domains of *TACC1* or *TACC3* (Supplementary Figure S1D). The distinctive feature of TACC proteins is a coiled-coil domain at the C terminus, known as the TACC domain, which exerts ligand-independent activation by dimerization and mediates localization to the mitotic spindle [44–46]. TACC3 plays a crucial role in the stabilization and organization of the mitotic spindle and thus proper chromosome segregation [44]. The *FGFR3-TACC3* fusion protein localizes to mitotic spindle poles, leading to mitotic and chromosomal segregation errors that contribute to aneuploidy in glioblastoma [11]. In addition, overexpression of FGFR is a consequence of formation of *FGFR3-TACC3* fusion due to loss of a suppressive effect of miR-99a on the 3′ UTR miRNA–binding site [47]. FGFR kinase inhibition by FGFR TKIs (PD173074, AZD4547, or BGJ398) prevents acquisition of aneuploidy defects [11]. Binding of FGF ligands to FGF receptors engages downstream signaling pathways, including PI3K-AKT and RAS-ERK, while the constitutively activated FGFR3-TACC3 protein promotes ERK and STAT3 signaling cascades in glioblastoma, suggesting acquisition of neomorphic functions [11, 47]. A truncated form of FGFR3 protein is not sufficient to cause kinase activation or transforming activity; thus the ability of the fusion protein [46] to dimerize is necessary for transformation. BAI1-associated protein 2–like 1 (also known as insulin receptor kinase substrate) is a fusion partner with FGFR3 in bladder cancer [46]. The FGFR3-BAIAP2L1 fusion promotes ligand-independent phosphorylation of FGFR3-BAIAP2L1 in Rat-2_F3-B cells and tumor formation in nude mice inoculated with Rat-2_F3-B cells [16] by activating ERK and STAT1 signaling [48], similar to *FGFR2-CCDC6* [48].

As lesson learned from RARA fusions in APL, with the product generated by the reciprocal translocations of RARA with multiple partners, contributed to the discovery of the biological response of APL to retinoids. Each RARA-containing fusion gene imparts a different treatment response, emphasizing the need to characterize fusion partners.

*RET* encodes a receptor tyrosine kinase protein containing an N-terminal extracellular domain with four cadherin-like repeats and a cysteine-rich region, a transmembrane domain, and C-terminal cytoplasmic tyrosine kinase domain. Wild-type RET is activated through ligand binding. Fusion proteins are activated through ligand-independent fusion of its intracellular kinase-encoding domain to coiled-coil domain containing 6 (*CCDC6*) or nuclear receptor coactivator 4 (*NCOA4*), among other partners. *RET* fusions mostly occur in irradiation-induced papillary thyroid carcinoma [49] as well as in lung adenocarcinoma. One common fusion partner in lung adenocarcinoma is *KIF5B.* The *RET-KIF5B* fusion retains a kinase domain from RET and a coiled-coil domain from KIF5B, which induces homodimerization and activates the oncogenic TK domain by autophosphorylation [50, 51]. Not only do the fusion proteins lead to dimerization but chimeric proteins lack the inhibitory domain of RET (Supplementary Table 1). Other partners of *RET* (Supplementary Figure 1F) include *TRIM24, TRIM27, ELKS, GOLGA5, PRKAR1A, RAB6IP2, MBD1, HOOK3, PCM1*, and *ERC1* in papillary thyroid carcinoma.

*A fourth mechanism* affects function through loss of pivotal domains: autoinhibitory segment, membrane, or nuclear localization domains. One example of this mechanism is *BRAF* fusion (Supplementary Table 2). *BRAF* encodes a protein called B-raf, a member of the RAF family of cytoplasmic serine/threonine protein kinases that contains an amino terminal RAS-binding domain and a carboxy-terminal kinase domain. BRAF and other RAF family members (*A-RAF* and *C-RAF*) are downstream effectors of activated RAS through activating the MEK-ERK signaling pathway and regulating multiple key functions of cells including growth, differentiation, apoptosis, and survival. Recently, BRAF fusions with different partners have been identified in a variety of epithelial tumors, including melanoma, pilocytic astrocytoma, papillary thyroid, rectal and prostate cancers. *SLC45A3-BRAF* (solute carrier family 45, member 3-v-raf murine sarcoma viral oncogene homolog B1) and *ESRP1-RAF1* (epithelial splicing regulatory protein-1–v-raf-1 murine leukemia viral oncogene homolog-1) gene fusions have been identified in prostate cancer, and *AGTRAP-BRAF* (encoding angiotensin II, type I receptor–associated protein–v-raf murine sarcoma viral oncogene homolog B1) has been found in gastric cancer [52]. SLC45A3 is a transmembrane transporter protein containing a transmembrane domain, and ESRP1 is a splicing factor containing RNA recognition and vascular domains. The expression of *SLC45A3-BRAF* or *ESRP1-RAF1* in prostate cells has been found to induce a neoplastic phenotype that is sensitive to RAF and MEK inhibitors [52]. The fusion proteins all lack the N-terminal RAS-binding domain and N-terminal autoinhibitory region of BRAF while retaining the kinase domain. Thus the fusion protein is constitutively active. In addition to the loss of the autoinhibitory region of BRAF, promoter regulatory elements from *SLC45A3*, which may be regulated by androgen (*SLC45A3-BRAF*) or promoter regulatory elements from *ESRP1*, lead to high expression of the chimeric transcript, which correlates with disease progression in prostate cancer metastases [53].

*PSF-TFE3* fusion occurs in papillary renal cell carcinoma [54]. TFE3 is a member of the class III helix-loop-helix (HLH) family, and PSF-A plays a role as a transcriptional repressor [55]. PSF-TFE3 fusion contains DBD and the dimerization domain (LZ region) of TFE3 and the repression domain of PSF, but this fusion lacks the N-terminal activation domain of TFE3 [54]. Parental TFE3 and PSF are nuclear proteins, whereas chimeric proteins localize in the endosomal compartment. Interestingly, the chimeric protein sequesters wild-type TFE3 and TP53 in the cytoplasm, leading to their degradation and to functionally null TP53 and TFE3, with subsequent transformation of cells [54].

*EWS-Oct-4* and *EWS-Oct-4B* fusions are common in sarcoma. OCT-4B encodes a nuclear protein that binds DNA with the same sequence specificity as the Oct-4 protein. Chimeric proteins comprise the N-terminal domain (NTD) of EWS and the POU and C-terminal domains of Oct-4 or Oct-4B. The POU domain is a conserved DNA-binding domain and mediates nuclear localization. EWS-Oct-4 and EWS-Oct-4B chimeric proteins localize in the nucleus, whereas Oct-4B mainly localizes in the cytoplasm. Both fusion proteins have transforming ability in nude mice [56, 57]. Another example is *RAD51C-ATXN7* fusion, which has been reported in 36% of colorectal cancers. The chimeric protein contains Rad51c N-terminal domains (ATP binding site and BRC (BRCA1) interacting domains) and the SCA7 (spino-cerebral ataxia 7) domain of ATXN7, and lacking C-terminal nuclear localization signal of Rad51C and CAG (polyglutamine tract) repeat sequence of ATXN7 [58]. This likely decreases the loading of Rad51 onto DNA during homologous recombination resulting in a shift to non-homologous end joining or persistence of double strand breaks resulting in increased genomic instability. *TRA2B-DNAH5* fusion has been found in the cytoplasm in lung squamous cell carcinoma (3.1%) and has transformation capacity in Ink4a-/- MEFs. It promotes cell invasion in Beas2B and CRL-5889 cells through activation of the MAPK pathway by decreasing the nuclear localization of SIRT6 [59]. It is important to note that localization of the protein itself is crucial for its role in cells. Even if the gene itself is not inactivated, changing the protein localization through fusion with other partners leads to degradation of the protein or an altered function in cells.

**Figure 3:**
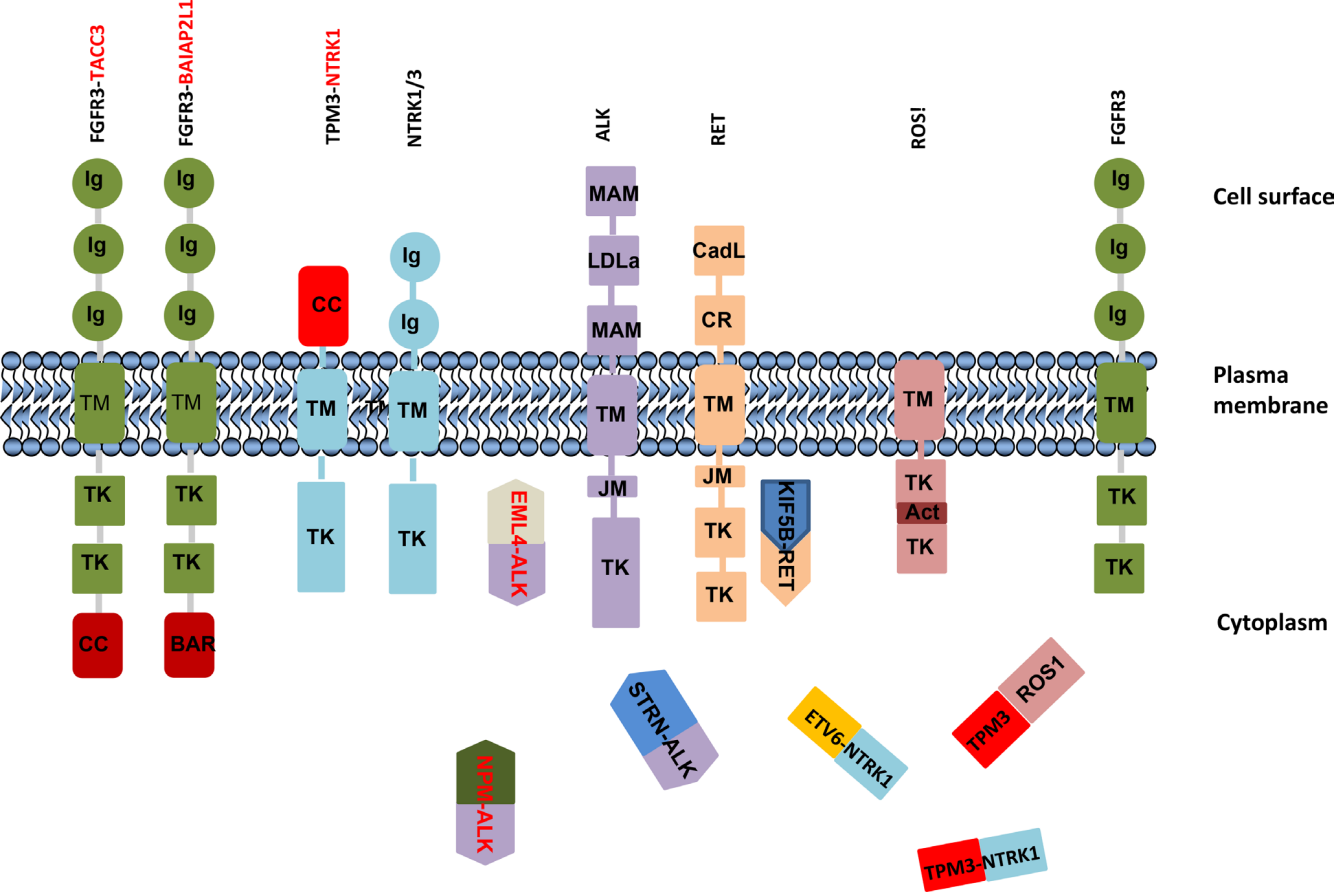
Schematic illustration of localization of parental and chimeric proteins

The *AKAP9-BRAF* fusion has been preferentially reported in radiation-induced papillary carcinomas, whereas the BRAFv600 mutation occurs in sporadic types of cancer [60]. The fusion protein contains the protein kinase domain but lacks the autoinhibitory N-terminal region of BRAF and the C-terminal centrosomal domain of AKAP9. The parental AKAP9 protein is found as a single dot in the centrosome (perinuclear) in normal cells, whereas the chimeric protein has a diffuse distribution in the cytoplasm. Chimeric protein is constitutively activated and has transforming ability in NIH3T3 cells and regulates the MAPK pathway [60]. In parental cells, however, growth factors, hormones, and cytokines regulate BRAF by binding and activating specific receptors on the cell surface [61]. Moreover, it is important to stress that chimeric protein formation may lead to loss of the transmembrane domain of protein and consequently to aberrant localization of chimeric proteins (Supplementary Table 1 and Figure 3).

*A fifth mechanism* impairs function by destroying the folding capacity of protein. MLL fusions occur in acute leukemia with a variety of partners (approximately 80). The breakpoints cluster mostly in MLL introns 9-11, with an age difference (in introns 9 and 10 in adults, and mostly in intron 11 in infants), with rare exceptional cases [62, 63]. MLL exons 11 to 16 code the PHD1-3 domains. When fusion breakpoints impair exon 11 in MLL, this results in the loss of two crucial cysteine residues, which prevents correct folding of the PHD domain, and influences its dimerization ability and binding capacity to its targets CYP33 and maybe to ECS^ASB2^ [64, 65]_._

*A sixth mechanism* affects function by the destruction of the regulatory role of a protein. Two scenarios are possible for this mechanism. Examples of the first scenario are the *AML1-ETO* and *PML-RARA* fusion genes. In this scenario, both fusion genes convert their transcription regulatory role from an ‘activator of gene transcription’ into a ‘repressor of transcription.’ Changing the transcription regulatory role of fusion genes depends on their interacting proteins, recruited corepressors or coactivators, and context-dependent cross talk with other proteins, which can bind to their target genes. An example of the second scenario is the *NAB2-STAT6* fusion, which converts the transcriptional repressor into a transcriptional activator in solitary fibrous tumor [66]. EGR1 induces NAB2, and NAB2 represses EGR1 by a negative feedback loop, while STAT is a transcriptional activator. In the context of solitary fibrous tumor, in *NAB2-STAT6* fusion, NAB2 inherits an activation domain from the STAT6, thus converting a ‘transcriptional repressor’ role into a ‘transcriptional activator’ of EGR1. This results in increasing cell proliferation by constitutive activation of EGR1, and through activation of EGR1 target genes (e.g., *NAB2*, *NAB1*, *IGF2*, *FGF2*, *PDGFD*, *FGFR1*, and *NTRK1*) rather than activation of STAT6 [66].

It is important to stress that multiple mechanisms may contribute to the transforming ability of fusion genes in cells. In *MYB-QKI* fusions, for example, the N-terminal HTH DNA-binding and transactivation domains of MYB and C-terminal region of QKI (QUA2 and Y rich) are retained, whereas the C-terminal negative regulatory domains of MYB and N-terminal KH RNA-binding domain of QKI are lost [67]. The nuclear localization sequence of QKI in C-terminal is retained in splice variant QKI5, with the protein being found in the nucleus, but nuclear localization sequence of QKI is lost in the splice variant QKI6 and it is likely found in the cytoplasm [68] Herein, MYB-QKI5 may also localize in nucleus, whereas MYB-QKI6 may localize in cytoplasm. It has been reported that 1621 and 1947 genes with the common 1029 genes are significantly differentially expressed in mouse neural stem cells that stably over-expressed MYB-QKI5 and MYB-QKI6 fusions compared to eGFP-expressing cells, respectively [67]. The distinct genes that differentially expressed may due to localization of these two splice variants. The oncogenic potential of MYB-QKI is mediated by multiple mechanisms. One possible mechanism can be through the autoregulatory feedback loop by binding the MYB-QKI fusion protein to the MYB promoter, resulting in its activation. The second mechanism is through loss of the N-terminal of QKI, resulting in downregulation of the tumor-suppressor activity of QKI. The third mechanism is through movement of the H3K27ac-bound enhancers in the 3′ region of QKI to the MYB promoter, resulting in activation of MYB [67]. The fourth mechanism is lack of negatively regulatory elements, miR15/miR16 binding sites of MYB are lost due to truncation of C-terminal domains of MYB. MYB belongs to a family of leucine zipper transcription factors and has a role in regulating cell proliferation, apoptosis, and differentiation [69]. Parental MYB lacks transforming ability *in vitro*, but C-terminus–truncated MYB proteins are oncogenic [70]. Destruction of MYB by fusion formation leads to disruption of target genes as well. QKI encodes the STAR (signal transduction and activation of RNA) RNA-binding protein Quaking, which has a pivotal role in oligodendroglial differentiation [71], and also has a role in regulating alternative splicing, microRNA processing, and circular RNA formation that involves in epithelial-mesenchymal transition [72–74]. *MYB-QKI* rearrangement occurs in most cases of angiocentric glioma (85.7%), and promote tumorigenesis both *in vitro* and *in vivo* [67]. The involvement of multiple mechanisms in transformation may be related to the aggressiveness of cancer and can contribute to patient prognosis.

## TARGETING FUSION GENES

It is important to stress that different gene partner domains or different breakpoints in fusion genes can alter the response to targeted therapy. Further, the intrinsic gene expression patterns in different tumors can also alter therapeutic sensitivity. As an example, CCDC6-RET and NCOA4-RET, despite both containing the same RET effector, drive different phenotypes and signaling resulting in differences in response to targeted therapeutics. Indeed, in model systems, different therapeutic approaches are necessary to optimally inhibit CCDC6-RET and NCOA4-RET [75].

Resistance to targeting fusion genes can occur through multiple mechanisms including gate-keeper mutations in the partner-kinase gene, amplification of the fusion gene or alternatively activating signaling pathways that can bypass the effects of the inhibitor (Supplementary Table 5). CCDC6-RET fusion expressing cells can become resistant to multi-targeted inhibitors such as sunitib or lenvatinib through EGFR activation and subsequent increased ERK-AKT signaling. Indeed, sunitib or lenvatinib resistant CCDC6-RET fusion expressing cells are resensitized by inhibition of the EGFR [76]. Similar processes have been observed in ALK, ROS, RET and NTRK1 fusion expressing cells [77, 78]. Importantly, intra-tumoral heterogeneity of fusion genes, similar to mutations, has been identified in tumors that could markedly alter the sensitivity to targeted therapeutics [79].

In addition, different fusion partners can affect the tumorigenicity of the fusion. For example, BAIAP2L1-MET and TFG-MET fusions are transforming, while CAPZA2-MET fusions are not transforming at least as assessed in Ba/F3 cells likely due to differences in subcellular localization [80]. Similarly, while FAM114A2-BRAF and ATG7-BRAF are transforming in Ba/F3 cells, AHCYL2-BRAF is not likely due to retention of an inhibitory domain within the N-terminus of BRAF [80].

It is important to note that in addition to altering responses to targeted therapy, gene fusions found in tumor cells can function as neoantigens, altering responses to immunotherapy [81]. Thus, immunotherapy could provide an alternative approach for therapy of some fusion gene containing tumors. Detailed experimental and modeling studies will be required to fully elucidate the effects of different partner genes, variable breakpoints, co-existence of other gene alterations, and tissue specific effects on sensitivity or resistance to targeted therapies.

A subset of oncogenic fusions can alter p53 activity and deregulate other check point and DNA-damage repair proteins. As noted above PFS-TFE3 fusions can increase degradation of p53. Furthermore, FGFR3-BAIAP2L1 fusions can suppress TP53 and CDKN2A expression as well as RB1 and p27 phosphorylation, associated with E2F2, E2F1, CDK2/Cyclin E and MAPK pathway activation [16]. Similarly, NPM-ALK JNK and MDM2-dependent inactivation of p53 function as well as PI3K-dependent p53 nuclear exclusion in anaplastic large cell lymphoma. Consistent with this concept inhibition of MDM2, JNK and PI3K induces apoptosis in NPM-ALK-expressing cells [82]. In addition, the MLL-ELL fusion inhibits functional activity of p53 in leukemia [83]. PLZF-RARa inhibits p53 and CDKN1A expression while increasing p53 degradation in acute promyelocytic leukemia [84]. Thus, p53 is a frequent target of fusion proteins.

In addition to p53, fusion proteins interact with other components of the DNA damage repair pathways. For example, BCR-ABL interacts with RAD51 resulting in stabilization and phosphorylation of RAD51 with subsequent increases in double strand break repair and drug resistance [85]. TMPRSS2-ERG fusion can block XRCC4-mediated NHEJ by inhibiting DNA-PKcs auto/trans-phosphorylation and thus sensitizes prostate cancer cells to PARP inhibition [86]. TMPRSS2-ERG and ETS family members can downregulate CHK1 expression and elevate DNA damage response in prostate tumor cells again potentially sensitizing cells to PARP inhibition [87].

Moreover, the effects of fusion genes can be both direct and indirect and are conditioned on co-occurring aberrations in other genes. For example, PTEN loss and p53 mutations frequently co-occur with ERG fusion proteins in prostate cancers [88–90]. In the presence of PTEN loss or p53 mutation, the effects of ERG fusion proteins would be modified. Notably expression of TMPRSS2(e1)-ERG(e4) in PC-3 cells inhibits expression a number of cell cycle-related genes including CDK1 and CCND1 thus promoting RB activation and E2F1 repression. This effect of ERG fusion proteins would interact with aberrations in either PTEN or p53. Thus, ERG fusion proteins alter the response of prostate cancer cells to palbociclib which has been demonstrated in human cell lines and mouse xenograft models [91].

## TUMOR TYPE–SPECIFIC FUSION TRANSCRIPTS

Fusion genes were at first thought to be specific for tumor types and hence useful as diagnostic and prognostic markers and for monitoring response to therapy. However, recent accumulated data has indicated that only a small portion of fusion genes are specific for certain types of cancer (Supplementary Table 4), and a number have been shown to occur in multiple tumor lineages. It is important to stress that even the same fusion gene can exhibit different oncogenic properties in different types of malignancies, for example, the breakpoints may differ from each other, as in *BCR-ABL* fusion genes. In this latter case, the fusion gene can be used as a diagnostic marker. This fusion gene is a defining marker of chronic myeloid leukemia (CML) (96%) and breakpoints in the majority of samples occur in the 5.8-kb major breakpoint cluster region (between introns 11 and 16), resulting in a p210 BCR-ABL protein. The same fusion gene with different breakpoints (minor breakpoint cluster region, within the 72-kb BCR intron 1) occurs in adult acute lymphocytic leukemia (30%), resulting in a p190 chimeric protein. It also occurs rarely in childhood ALL (3-5%) and acute myeloid leukemia (1%) [3]. Defining fusion genes have been reported in epithelial tumors as well. *ESRRA-C11orf20* fusion gene has been found in a subset of serous ovarian cancers (15%) [92], while *EIF3E–RSPO2* and *PTPRK–RSPO3* fusion genes (in which chimeric protein activate Wnt signaling) have been found in 10% of colorectal cancers [7]. Other examples of defining fusions are *SLC3A2-NRG1* (in which chimeric protein activates AKT and ERK pathways) in invasive mucinous adenocarcinoma of the lung (27%) [93], and *EGFR-SEPT14* fusion gene in glioblastoma [94]. *JMJD7-PLA2G4B* has been reported in HNSCC [95], and chimeric protein promotes cell proliferation and survival by modulating phosphorylation of AKT and regulates cell cycle progression by regulating SKP2 in HNSCC. PLAG2G4B encodes a calcium-dependent phospholipase that hydrolyzes phospholipids to lysophospholipids and fatty acids with lysophospholipids being potent signaling molecules [95]. *DNAJB1-PRKACA* fusion is a defining biomarker in fibrolamellar hepatocellular carcinoma (100%) and has not been seen in other subtypes of hepatocellular carcinoma [96]. DNAJB1 encodes a heat shock protein, Hsp40, which is involved in protein folding within cells [97]. A chimeric protein contains the N-terminal of DNAJB1 and the C-terminal of PRKACA. Parental PRKACA involves glucose and lipid metabolism and mitochondrial biogenesis [96, 98, 99]. *BRD-NUT* fusions have been reported in NUT midline carcinoma (66%) [100, 101], and *MAML2* fusions—*CRTC3-MAML2* (<1%) and *CRC1-MAML2* (30–75%)—have been reported in mucoepidermoid carcinoma (MEC) [102, 103], in infantile lung MEC [104], in cervix [105], in thyroid and salivary glands and Warthin’s tumor [106], as well as in hidradenoma of the breast parenchyma [107]. Subsets of breast cancer and head and neck cancer are characterized by MYB-NFIB gene fusions (Supplementary Figure 1I) [32]. *TFE3-TFEB* fusions are characteristic of renal cell carcinoma [108]. *ETV6-NTRK3* has been found in infantile fibrosarcoma, secretory breast carcinoma (>90%) [109], and acute myeloid leukemia [110]. As a result of this fusion, transforming occurs through activation of RAS-MAPK and PI3K-AKT and AP1 transcription complex [111]. *MTAP-ANRIL* fusion has been reported in melanoma [112] and *CLDN18-ARHGAP26* in gastric cancer, the latter promoting loss of the epithelial phenotype in gastric cancer [113].*TMPRSS2* fusions may be defining markers for prostate cancer. *MYB-QKI* fusion genes are defined biomarkers in angiocentric glioma [67]. Of note, *ESR1-YAP* and *ESR1-PCDH11X* fusions have crucial role in resistance to endocrine therapy, and trigger metastasis to lung in ER-positive breast cancer [114].

## FUSION GENES COMMON IN MULTIPLE TYPES OF CANCER

In contrast, a number of TK fusion genes, such as *ALK, ROS1, FGFR, NTRK*, and *RET*, have been identified in multiple cancer lineages. The *ALK* gene rearrangement was first identified in anaplastic large cell lymphoma (50%) in 1994 [115, 116]. Soon afterwards, this rearrangement was identified in NSCLC [117, 118] papillary thyroid cancer, colorectal cancer [12, 13, 119], renal cell cancer [120], and esophageal [121], breast, and gastric cancers and acute myeloid leukemia [117], as well as in spitzoid tumors (10%) [122] (Supplementary Table 1). *ALK*’s most common fusion partners are the *NPM1* gene in anaplastic large cell lymphoma [115] and *EML4* in epithelial tumors. *EML4-ALK* fusion gene occurs mostly in young NSCLC patients who are non-smokers or light smokers, and it is more common among patients of Asian ancestry (7.4%) than among patients of European ancestry (3.0%) [12, 117, 123]. Similar to ALK fusions, FGFR fusions have been reported in a wide range of tumors (cholangiocarcinoma, breast cancer, prostate cancer, NSCLC, gastric adenocarcinoma, colorectal adenocarcinoma, carcinoma of unknown primary, and glioblastoma) with a variety of partners, including *TACC3, PPAPDC1A*, *AFF3*, *SLC45A3* and *AHCYL1, C10orf68, JAKMIP1, KIAA1598*, *NCALD*, *NOL4*, *NTM*, *PPAPDCA*, *TNIP2*, and *WHSC1* [12, 13, 117, 119, 123]. Indeed, *ROS1* fusion was first identified in 1987 in a glioblastoma multiforme cell line [124]. Over the last two decades, discovery of *ROS1* fusion has followed in lung adenocarcinoma (1.0-2.5%) [125], gastric cancer (0.61%), cholangiocarcinoma including biliary tract carcinoma (3.9%), colon cancer (0.85%), Spitz nevus (benign) (25.3%), atypical Spitz tumors (6.2%), and spitzoid melanomas (9.1%) [122, 126–130]. These findings raise the possibility that breakpoints may differ among the tumor types or downstream signaling pathways. On the other hand, some of the partner genes are common among fusions: *CD74-NRG1* and *CD74-ROS1*, *KIF5B-ALK* and *KIF5B-RET*, *TPM3-ALK* and *TPM3-NTRK1* (Supplementary Figure 1C, 1F–1H) [93].

## ARE FUSION GENES THE CAUSE OR CONSEQUENCE OF TUMORIGENESIS?

The causes of structural rearrangements remain poorly understood. As aforementioned DNA rearrangements resulting in fusion genes can be triggered by multiple factors, including cellular stress, inappropriate repair or recombination of DNA (e.g., antigen receptor diversity-generating enzymes, homologous recombination, and non-homologous end joining), DNA sequence and chromatin features (e.g., chromatin modification, repetitive elements, CpG dinucleotides, non-B DNA structure), and spatial proximity (the distance between an oncogene and its translocation partner) [131]. Cellular stress includes genotoxic, oxidative, replication, and transcriptional stresses. Exposure to radiation or chemical compounds such as alkylating agents can lead to genotoxic stress affecting genome stability [131–133]. A high level of reactive oxygen species is also associated with genomic rearrangements [131]. Replication stress is associated with inefficient DNA replication, due to multiple factors. These factors include imbalance of DNA replication enzymes, low level of folate, presence of low-copy number repeats, or fragile sites [131, 134]. Replication stress can also be caused by inhibition of DNA polymerases such as that induced by aphidicolin [131]. The most prevalent examples of fusion genes caused by radiation-induced genotoxic stress are *RET fusions* found in irradiation-induced papillary thyroid carcinoma [135]. Dietary bioflavonoids, which are a component of natural foods and dietary supplements, cause site-specific DNA cleavage in the mixed-lineage leukemia (MLL) breakpoint cluster region *in vivo,* representing the most prominent example of fusion genes caused by chemical component–induced genotoxic stress [136]. Bioflavonoid-induced DNA breaks may be due to inhibition of topoisomerase II [136]. Treatment with topoisomerase II inhibitors can lead to double-stand breaks and MLL gene translocation in acute leukemia patients as well as to the development of secondary leukemia [137, 138]. Topoisomerases can both cause and repair double-strand breaks, indicating that the toxicity of current cancer treatments may also cause the formation of fusion genes and the development of secondary malignancies or resistance to therapy. The interaction of androgen receptor (genotoxic stress) and activation-induced cytidine deaminase (AID) (transcriptional stress) is likely to be the cause of the double-strand breaks that drive the specific changes in the genome and/or the chromatin remodeling that bring a *TMPRSS2* into proximity with *ETS* genes to form gene fusions (*TMPRSS2-ERG*, *TMPRSS2-ETV1*, and *SLC45A3-ETV1)* in prostate cancer [24, 139, 140].

## FUSION GENES IN NORMAL EPITHELIAL CELLS, DEVELOPMENTAL DISEASES, AND BENIGN TUMORS

Fusion genes occur mostly in tumor cells and have been implicated in tumorigenesis, progression or resistance to therapy. They also occur in benign tumors [122] and developmental disorders [141], as well as in normal cells [142]. One example is the *JAZF1-JJAZ1* fusion, which occurs through trans-splicing in normal endometrial stromal cells [143] but not in other cells. The same chimeric gene has been observed in human endometrial stromal tumors [144]. Multiple read-through fusion transcripts, *ELAVL1-TIMM44, FAM162B-ZUFSP, IFNAR2-IL10RB, INMT-FAM188B, KIAA1841-C2orf74, NFATC3-PLA2G15, SIRPB1-SIRPD,* and *SHANK3-ACR*, also have been found in normal lung tissue from patients with lung adenocarcinoma [142]. In addition, *YPEL5-PPP1CB* fusion transcript has been detected in normal samples and in different hematologic malignancies [145]. Notably, fusion genes may be more common in normal epithelial cells than was at first thought. *ADAMTS6-ARID1B* or *ARID1B-ADAMTS6* fusion gene formation occurs by translocation in patients with developmental delay; however, no fusion transcripts were formed due to the opposing transcriptional direction in both genes. However, the formation of fusion led to a small deletion in *ARID1B*, which may be a cause of developmental delay [141]. ADAMTS6 encodes a zinc metalloprotease, and ARID1B (AT-rich interactive domain 1B (SWI-1-like) encodes the DNA-binding subunit of the SWI/SNF chromatin-remodeling complex, which is involved in DNA replication, repair, and transcriptional regulation [146]. Even if the fusion gene lacks a chimeric transcript or protein, it still may be important. Other changes may occur during the fusion formation, such as deletion or duplication of part of a gene. Therefore, such a gene may still be relevant in development of complex diseases including cancers.

## CONCLUSION AND FUTURE DIRECTIONS

It has been known that oncogenic fusion genes influence tumorigenesis through a number of different mechanisms. However, much work remains in elucidating the role of chimeric proteins in tumorigenesis. Fusion genes can compromise transcription factors, tyrosine kinases, metabolic pathways, DNA repair, and signaling pathways such as Wnt. Fusion genes that disrupt transcription factor genes can result in chimeric proteins that have enhanced, repressed, or aberrant transcriptional activity. Fusions that involve transcription factors retain the DNA-binding domain of the transcription factor and have a potent transactivation or suppression motif that induces or suppresses the transcription of target genes. This mediates tumor growth, suppresses target genes needed for normal cell differentiation, and contributes to the accumulation of immature cells. Fusion genes that disrupt tyrosine kinase can result in chimeric proteins with aberrant tyrosine kinase activity promoting downstream signaling pathways. In many kinases, the TK domain locates in the C-terminus, whereas an inhibitory domain that inactivates the kinase activity is found in the N-terminus. In many fusion proteins, the partner gene replaces the N-terminal portion of the protein, while the C-terminal TK domain is retained. Additionally, expression of the fusion product is controlled by the promoter of the partner gene. The activation of chimeric proteins that contain tyrosine kinase is due to dimerization or oligomerization domain of partner genes, which is essential for cell transformation. For example, coiled-coil domains in *EML4, C20rf44, KIF5B, CCDC6, NCOA4, TACC3*, and *TACC1* are fused to kinase domains of *ALK*, *RET, FGFR3, or FGFR1* in *EML4-ALK, C2orf44-ALK, KIF5B-RET, CCDC6-RET, NCOA4-RET, FGFR3-TACC3*, and *FGFR1-TACC1* fusion genes, respectively. As a result, the fusion protein contains a protein kinase domain and a coiled-coil domain with a lack of an inactivation segment. The coiled-coil domain confers ligand-independent dimerization and oligomerization on the fusion kinase, resulting in autophosphorylation-induced constitutive kinase activity. In these cases, inhibiting the dimerization domain with drugs such as stapled peptides may provide an effective strategy for inhibiting the tyrosine kinase activity of the fusion protein. On the other hand, targeting the other distracted genes or pathways such as DNA repair and metabolic pathways may be considered in the treatment of disease. Effective, non-toxic agents that block only the rearranged protein but not normal ones are urgently needed. Identifying the factors that cause gene fusions will improve our understanding of cancer pathogenesis, lead to the development of effective anticancer agents, and help in developing cancer-preventive agents. Defining markers can serve as diagnostic biomarkers in cancer patients.

A number of questions remain to be answered. Do fusion genes activate the same downstream signaling pathways and cause the same clinical outcome in the different types of epithelial tumors? Do all fusion partners across diseases have the same response to therapeutic agents? Current knowledge about fusion genes and their effect in tumorigenesis remains only the tip of the iceberg, and there is still much more to discover.

## SUPPLEMENTARY MATERIALS FIGURE AND TABLES






