# Supplementary material for: Molecular mechanisms and pathobiology of oncogenic fusion transcripts in epithelial tumors
Source: Oncotarget. 2019 Mar 12;10(21):2095–111. doi: 10.18632/oncotarget.26777 (PMC6459343; doi:10.18632/oncotarget.26777)
Supplement: Supplementary file 1 [file oncotarget-10-2095-s001.pdf]

Molecular mechanisms and pathobiology of oncogenic fusion transcripts in epithelial tumors

SUPPLEMENTARY MATERIALS

Supplementary Table 1: Examples of oncogenic fusion genes of receptor tyrosine kinases in epithelial tumors. See Supplementary_Table_1

Supplementary Table 2: Examples of oncogenic fusion genes of serine/threonine kinases in epithelial tumors

Fusion gene	Tumor type	Type of rearrangements	Fusion gene domains	Ref
BRAF partners				
KIAA1549	PA (70%), ADG (9.4%)	DEL, TD	N-PK	[52]
SND1	PACC, gastric cancer, SCM, LUAD never smokers	INV	$TN_{(2–3)}$-PK (pancreatic acinar)	[45, 53, 54]
			TN3-RBD-CRD-PK (LUAD)	[52]
PAPSS1	SCM, rectal carcinoma	TR	APS-PK	[53]
TRIM24	Pilocytic astrocytoma	INV	BRM-PK	[53]
FAM131B	Pilocytic astrocytoma	DEL	N-PK	[52, 55]
RNF130	Pilocytic astrocytoma	TR	N-PK	[49]
MKRN1	Pilocytic astrocytoma, anaplastic thyroid cancer	TD	N-PK	[49, 56]
SLC45A3	Prostate cancer	INV	N-CR-PK	[57]
AKAP9	PTC, melanocytic nevi	INV	N-PK	[55, 58]
AGTRAP	Gastric cancer	TR	N-PK	[55]
FCHSD1	Melanocytic nevi	TR	BAR-PK	[56]
RAF1 partners				
SRGAP3	Pilocytic astrocytoma, prostate cancer	TD	N-PK	[57]
ESRP1	Prostate cancer	TR	N-PK	[57]
ATG7	Pancreatic cancer	DEL	N-PK	[55]
BCL6	Anaplastic astrocytoma	INV	N-PK	[55]
NFIA	Pilocytic astrocytoma	TR	N-PK	[59]

N, N-terminal part of the partner protein of chimeric protein.

Supplementary Table 3: Examples of oncogenic fusion genes of transcription factors or regulators

Fusion genes	Tumor types (%)	Type of rearrangements	Refs
TMPRSS2-ERG[#]	PCa (~50%)	DEL, INV	[60–62]
TMPRSS2-ETV1	PCa (5–10%)	TR	[19, 25, 26, 28, 29, 61–63]
TMPRSS2-ETV4	PCa (<5%)	TR	[64]
TMPRSS2-ETV5	PCa (<5%)	TR	[65]
ACSL3-ETV1	PCa	TR	[66]
FLJ35294-ETV1	PCa	TR	[67]
DDX5-ETV4	PCa	INV	[67]
KLK2-ETV4	PCa	TR	[68]
CANT1-ETV4	PCa	INV	[68]
SLC45A3-ERG	PCa	TR	[61]
C15orf21-ETV1	PCa	TR	[61]
HNRPA2B1-ETV1	PCa	DEL	[61]
SLC45A3-ETV1	PCa	TR	[65]
SLC45A3-ETV5	PCa	TR	[61]
HERVK22q11.23	PCa	DEL	[69]
SLC45A3-FLI1	PCa	TR	[69]
PVT1-MYC<br>*PVT1-NDRG1*	Medulloblastoma	Chromothripsis<br>INV	[19, 22, 25, 26, 29, 70]
NFIB-MYB<br>*QKI-MYB*<br>*HMGIC-MYB*	HNACC (86%), ACCB (67%), BACC,<br>SGC (52.9%), ACCSG (28%)<br>Angiocentric glioma (PLGG) (85.7%)<br>Pleomorphic adenoma	TR<br>DEL	[71–76]<br>[77]<br>[71]

Supplementary Table 4: Examples of specific fusion genes of defined tumors

Fusion genes	Tumor types (%)	Type of rearrangement	Ref
EIF3E-RSPO2	Colon cancer (0–5%)	DEL	[78]
RTPRK-RSPO3	Colon cancer (5–10%)	INV	
ESRRA-C11orf20	Serous ovarian cancer (15%)	TD	[79]
SLC3A2-NRG1	Invasive mucinous adenocarcinoma of the lung (27%)	TR	[80]
JMJD7-PLA2G4B	HNSCC	RT	[81]
DNAJB1-PRKACA	Fibrolamellar HCC (100%)	DEL	[82]
BRD-NUT	NUT midline carcinoma (66%)	TR	[83]
TFE3-TFEB	Renal cell carcinoma	TR	[84, 85]
MTAP-ANRIL	Melanoma	DEL	[86]
CLDN18-ARHGAP26	Gastric cancer	TR	[87]
RAD51-ATXN7	Colorectal cancer	TR	[88]

Supplementary Table 5: Examples of oncogenic fusions that sensitive or resistant to targeted therapeutics agents. See Supplementary_Table_5

REFERENCES

1. Soda M, Choi YL, Enomoto M, Takada S, Yamashita Y, Ishikawa S, Fujiwara S, Watanabe H, Kurashina K, Hatanaka H, Bando M, Ohno S, Ishikawa Y, et al. Identification of the transforming EML4-ALK fusion gene in non-small-cell lung cancer. Nature. 2007; 448:561–66.
2. Aisner DL, Nguyen TT, Paskulin DD, Le AT, Haney J, Schulte N, Chionh F, Hardingham J, Mariadason J, Tebbutt N, Doebele RC, Weickhardt AJ, Varella-Garcia M. ROS1 and ALK fusions in colorectal cancer, with evidence of intratumoral heterogeneity for molecular drivers. Mol Cancer Res. 2014; 12:111–18.
3. Lin E, Li L, Guan Y, Soriano R, Rivers CS, Mohan S, Pandita A, Tang J, Modrusan Z. Exon array profiling detects EML4-ALK fusion in breast, colorectal, and non-small cell lung cancers. Mol Cancer Res. 2009; 7:1466–76.
4. Lipson D, Capelletti M, Yelensky R, Otto G, Parker A, Jarosz M, Curran JA, Balasubramanian S, Bloom T, Brennan KW, Donahue A, Downing SR, Frampton GM, et al. Identification of new ALK and RET gene fusions from colorectal and lung cancer biopsies. Nat Med. 2012; 18:382–84.
5. Hernández L, Pinyol M, Hernández S, Beà S, Pulford K, Rosenwald A, Lamant L, Falini B, Ott G, Mason DY, Delsol G, Campo E. TRK-fused gene (TFG) is a new partner of ALK in anaplastic large cell lymphoma producing two structurally different TFG-ALK translocations. Blood. 1999; 94:3265–68.
6. Takeuchi K, Choi YL, Togashi Y, Soda M, Hatano S, Inamura K, Takada S, Ueno T, Yamashita Y, Satoh Y, Okumura S, Nakagawa K, Ishikawa Y, Mano H. KIF5B-ALK, a novel fusion oncokinase identified by an immunohistochemistry-based diagnostic system for ALK-positive lung cancer. Clin Cancer Res. 2009; 15:3143–49.
7. Togashi Y, Soda M, Sakata S, Sugawara E, Hatano S, Asaka R, Nakajima T, Mano H, Takeuchi K. KLC1-ALK: a novel fusion in lung cancer identified using a formalin-fixed paraffin-embedded tissue only. PLoS One. 2012; 7:e31323.
8. Morris SW, Kirstein MN, Valentine MB, Dittmer KG, Shapiro DN, Saltman DL, Look AT. Fusion of a kinase gene, ALK, to a nucleolar protein gene, NPM, in non-Hodgkin's lymphoma. Science. 1994; 263:1281–84.
9. Debelenko LV, Raimondi SC, Daw N, Shivakumar BR, Huang D, Nelson M, Bridge JA. Renal cell carcinoma with novel VCL-ALK fusion: new representative of ALK-associated tumor spectrum. Mod Pathol. 2011; 24:430–42.
10. Du XL, Hu H, Lin DC, Xia SH, Shen XM, Zhang Y, Luo ML, Feng YB, Cai Y, Xu X, Han YL, Zhan QM, Wang MR. Proteomic profiling of proteins dysregulted in Chinese esophageal squamous cell carcinoma. J Mol Med (Berl). 2007; 85:863–75.
11. Ma Z, Hill DA, Collins MH, Morris SW, Sumegi J, Zhou M, Zuppan C, Bridge JA. Fusion of ALK to the Ran-binding protein 2 (RANBP2) gene in inflammatory myofibroblastic tumor. Genes Chromosomes Cancer. 2003; 37:98–105.
12. Kelly LM, Barila G, Liu P, Evdokimova VN, Trivedi S, Panebianco F, Gandhi M, Carty SE, Hodak SP, Luo J, Dacic S, Yu YP, Nikiforova MN, et al. Identification of the transforming STRN-ALK fusion as a potential therapeutic target in the aggressive forms of thyroid cancer. Proc Natl Acad Sci USA. 2014; 111:4233–38.
13. Yakirevich E, Resnick MB, Mangray S, Wheeler M, Jackson CL, Lombardo KA, Lee J, Kim KM, Gill AJ, Wang K, Gowen K, Sun J, Miller VA, et al. Oncogenic alk fusion in rare and aggressive subtype of colorectal adenocarcinoma as a potential therapeutic target. Clin Cancer Res. 2016; 22:3831–40.
14. Singh D, Chan JM, Zoppoli P, Niola F, Sullivan R, Castano A, Liu EM, Reichel J, Porrati P, Pellegatta S, Qiu K, Gao Z, Ceccarelli M, et al. Transforming fusions of FGFR and TACC genes in human glioblastoma. Science. 2012; 337:1231–35.
15. Williams SV, Hurst CD, Knowles MA. Oncogenic FGFR3 gene fusions in bladder cancer. Hum Mol Genet. 2013; 22:795–803.
16. Wang R, Wang L, Li Y, Hu H, Shen L, Shen X, Pan Y, Ye T, Zhang Y, Luo X, Zhang Y, Pan B, Li B, et al. FGFR1/3 tyrosine kinase fusions define a unique molecular subtype of non-small cell lung cancer. Clin Cancer Res. 2014; 20:4107–14.
17. Wu YM, Su F, Kalyana-Sundaram S, Khazanov N, Ateeq B, Cao X, Lonigro RJ, Vats P, Wang R, Lin SF, Cheng AJ, Kunju LP, Siddiqui J, et al. Identification of targetable FGFR gene fusions in diverse cancers. Cancer Discov. 2013; 3:636–47.
18. Arai Y, Totoki Y, Hosoda F, Shirota T, Hama N, Nakamura H, Ojima H, Furuta K, Shimada K, Okusaka T, Kosuge T, Shibata T. Fibroblast growth factor receptor 2 tyrosine kinase fusions define a unique molecular subtype of cholangiocarcinoma. Hepatology. 2014; 59:1427–34.
19. Banerji S, Cibulskis K, Rangel-Escareno C, Brown KK, Carter SL, Frederick AM, Lawrence MS, Sivachenko AY, Sougnez C, Zou L, Cortes ML, Fernandez-Lopez JC, Peng S, et al. Sequence analysis of mutations and translocations across breast cancer subtypes. Nature. 2012; 486:405–09.
20. Chao BH, Briesewitz R, Villalona-Calero MA. RET fusion genes in non-small-cell lung cancer. J Clin Oncol. 2012; 30:4439–41.
21. Grieco M, Santoro M, Berlingieri MT, Melillo RM, Donghi R, Bongarzone I, Pierotti MA, Della Porta G, Fusco A, Vecchio G. PTC is a novel rearranged form of the ret proto-oncogene and is frequently detected *in vivo* in human thyroid papillary carcinomas. Cell. 1990; 60:557–63.
22. Kandoth C, McLellan MD, Vandin F, Ye K, Niu B, Lu C, Xie M, Zhang Q, McMichael JF, Wyczalkowski MA, Leiserson MD, Miller CA, Welch JS, et al. Mutational

landscape and significance across 12 major cancer types. Nature. 2013; 502:333–39.

23. Kohno T, Ichikawa H, Totoki Y, Yasuda K, Hiramoto M, Nammo T, Sakamoto H, Tsuta K, Furuta K, Shimada Y, Iwakawa R, Ogiwara H, Oike T, et al. KIF5B-RET fusions in lung adenocarcinoma. Nat Med. 2012; 18:375–77.
24. Takeuchi K, Soda M, Togashi Y, Suzuki R, Sakata S, Hatano S, Asaka R, Hamanaka W, Ninomiya H, Uehara H, Lim Choi Y, Satoh Y, Okumura S, et al. RET, ROS1 and ALK fusions in lung cancer. Nat Med. 2012; 18:378–81.
25. Bell D, Berchuck A, Birrer M, Chien J, Cramer DW, Dao F, Dhir R, DiSaia P, Gabra H, Glenn P, Godwin AK, Gross J, Hartmann L, et al, and Cancer Genome Atlas Research Network. Integrated genomic analyses of ovarian carcinoma. Nature. 2011; 474:609–15.
26. Cancer Genome Atlas Network. Comprehensive molecular portraits of human breast tumours. Nature. 2012; 490:61–70.
27. Cancer Genome Atlas Research Network. Comprehensive genomic characterization of squamous cell lung cancers. Nature. 2012; 489:519–25.
28. Cancer Genome Atlas Network. Comprehensive molecular characterization of human colon and rectal cancer. Nature. 2012; 487:330–37.
29. Cancer Genome Atlas Research Network. Comprehensive molecular characterization of clear cell renal cell carcinoma. Nature. 2013; 499:43–49.
30. Bongarzone I, Butti MG, Coronelli S, Borrello MG, Santoro M, Mondellini P, Pilotti S, Fusco A, Della Porta G, Pierotti MA. Frequent activation of ret protooncogene by fusion with a new activating gene in papillary thyroid carcinomas. Cancer Res. 1994; 54:2979–85.
31. Drilon A, Wang L, Hasanovic A, Suehara Y, Lipson D, Stephens P, Ross J, Miller V, Ginsberg M, Zakowski MF, Kris MG, Ladanyi M, Rizvi N. Response to Cabozantinib in patients with RET fusion-positive lung adenocarcinomas. Cancer Discov. 2013; 3:630–35.
32. Fugazzola L, Pilotti S, Pinchera A, Vorontsova TV, Mondellini P, Bongarzone I, Greco A, Astakhova L, Butti MG, Demidchik EP. Oncogenic rearrangements of the RET proto-oncogene in papillary thyroid carcinomas from children exposed to the Chernobyl nuclear accident. Cancer Res. 1995; 55:5617–20.
33. Salassidis K, Bruch J, Zitzelsberger H, Lengfelder E, Kellerer AM, Bauchinger M. Translocation t(10;14)(q11.2:q22.1) fusing the kinetin to the RET gene creates a novel rearranged form (PTC8) of the RET proto-oncogene in radiation-induced childhood papillary thyroid carcinoma. Cancer Res. 2000; 60:2786–89.
34. Awad MM, Katayama R, McTigue M, Liu W, Deng YL, Brooun A, Friboulet L, Huang D, Falk MD, Timofeevski S, Wilner KD, Lockerman EL, Khan TM, et al. Acquired resistance to crizotinib from a mutation in CD74-ROS1. N Engl J Med. 2013; 368:2395–401.
35. Rikova K, Guo A, Zeng Q, Possemato A, Yu J, Haack H, Nardone J, Lee K, Reeves C, Li Y, Hu Y, Tan Z, Stokes M, et al. Global survey of phosphotyrosine signaling identifies oncogenic kinases in lung cancer. Cell. 2007; 131:1190–203.
36. Yoshida A, Kohno T, Tsuta K, Wakai S, Arai Y, Shimada Y, Asamura H, Furuta K, Shibata T, Tsuda H. ROS1-rearranged lung cancer: a clinicopathologic and molecular study of 15 surgical cases. Am J Surg Pathol. 2013; 37:554–62.
37. Lee J, Lee SE, Kang SY, Do IG, Lee S, Ha SY, Cho J, Kang WK, Jang J, Ou SH, Kim KM. Identification of ROS1 rearrangement in gastric adenocarcinoma. Cancer. 2013; 119:1627–35.
38. Wiesner T, He J, Yelensky R, Esteve-Puig R, Botton T, Yeh I, Lipson D, Otto G, Brennan K, Murali R, Garrido M, Miller VA, Ross JS, et al. Kinase fusions are frequent in Spitz tumours and spitzoid melanomas. Nat Commun. 2014; 5:3116.
39. Charest A, Lane K, McMahon K, Park J, Preisinger E, Conroy H, Housman D. Fusion of FIG to the receptor tyrosine kinase ROS in a glioblastoma with an interstitial del(6)(q21q21). Genes Chromosomes Cancer. 2003; 37:58–71.
40. Suehara Y, Arcila M, Wang L, Hasanovic A, Ang D, Ito T, Kimura Y, Drilon A, Guha U, Rusch V, Kris MG, Zakowski MF, Rizvi N, et al. Identification of KIF5B-RET and GOPC-ROS1 fusions in lung adenocarcinomas through a comprehensive mRNA-based screen for tyrosine kinase fusions. Clin Cancer Res. 2012; 18:6599–608.
41. Rimkunas VM, Crosby KE, Li D, Hu Y, Kelly ME, Gu TL, Mack JS, Silver MR, Zhou X, Haack H. Analysis of receptor tyrosine kinase ROS1-positive tumors in non-small cell lung cancer: identification of a FIG-ROS1 fusion. Clin Cancer Res. 2012; 18:4449–57.
42. Bergethon K, Shaw AT, Ou SH, Katayama R, Lovly CM, McDonald NT, Massion PP, Siwak-Tapp C, Gonzalez A, Fang R, Mark EJ, Batten JM, Chen H, et al. ROS1 rearrangements define a unique molecular class of lung cancers. J Clin Oncol. 2012; 30:863–70.
43. Peraldo Neia C, Cavalloni G, Balsamo A, Venesio T, Napoli F, Sassi F, Martin V, Frattini M, Aglietta M, Leone F. Screening for the FIG-ROS1 fusion in biliary tract carcinomas by nested PCR. Genes Chromosomes Cancer. 2014; 53:1033–40.
44. Govindan R, Ding L, Griffith M, Subramanian J, Dees ND, Kanchi KL, Maher CA, Fulton R, Fulton L, Wallis J, Chen K, Walker J, McDonald S, et al. Genomic landscape of non-small cell lung cancer in smokers and never-smokers. Cell. 2012; 150:1121–34.
45. Fujimoto A, Totoki Y, Abe T, Boroevich KA, Hosoda F, Nguyen HH, Aoki M, Hosono N, Kubo M, Miya F, Arai Y, Takahashi H, Shirakihara T, et al. Whole-genome sequencing of liver cancers identifies etiological influences

on mutation patterns and recurrent mutations in chromatin regulators. Nat Genet. 2012; 44:760–64.

46. Kobayashi S, Boggon TJ, Dayaram T, Jänne PA, Kocher O, Meyerson M, Johnson BE, Eck MJ, Tenen DG, Halmos B. EGFR mutation and resistance of non-small-cell lung cancer to gefitinib. N Engl J Med. 2005; 352:786–92.
47. Cancer Genome Atlas Research Network. Comprehensive genomic characterization defines human glioblastoma genes and core pathways. Nature. 2008; 455:1061–68.
48. Tognon C, Knezevich SR, Huntsman D, Roskelley CD, Melnyk N, Mathers JA, Becker L, Carneiro F, MacPherson N, Horsman D, Poremba C, Sorensen PH. Expression of the ETV6-NTRK3 gene fusion as a primary event in human secretory breast carcinoma. Cancer Cell. 2002; 2:367–76.
49. Jones DT, Hutter B, Jäger N, Korshunov A, Kool M, Warnatz HJ, Zichner T, Lambert SR, Ryzhova M, Quang DA, Fontebasso AM, Stütz AM, Hutter S, et al, and International Cancer Genome Consortium PedBrain Tumor Project. Recurrent somatic alterations of FGFR1 and NTRK2 in pilocytic astrocytoma. Nat Genet. 2013; 45:927–32.
50. Greco A, Pierotti MA, Bongarzone I, Pagliardini S, Lanzi C, Della Porta G. TRK-T1 is a novel oncogene formed by the fusion of TPR and TRK genes in human papillary thyroid carcinomas. Oncogene. 1992; 7:237–42.
51. Vaishnavi A, Capelletti M, Le AT, Kako S, Butaney M, Ercan D, Mahale S, Davies KD, Aisner DL, Pilling AB, Berge EM, Kim J, Sasaki H, et al. Oncogenic and drug-sensitive NTRK1 rearrangements in lung cancer. Nat Med. 2013; 19:1469–72.
52. Cin H, Meyer C, Herr R, Janzarik WG, Lambert S, Jones DT, Jacob K, Benner A, Witt H, Remke M, Bender S, Falkenstein F, Van Anh TN, et al. Oncogenic FAM131B-BRAF fusion resulting from 7q34 deletion comprises an alternative mechanism of MAPK pathway activation in pilocytic astrocytoma. Acta Neuropathol. 2011; 121:763–74.
53. Jones DT, Kocialkowski S, Liu L, Pearson DM, Ichimura K, Collins VP. Oncogenic RAF1 rearrangement and a novel BRAF mutation as alternatives to KIAA1549:BRAF fusion in activating the MAPK pathway in pilocytic astrocytoma. Oncogene. 2009; 28:2119–23.
54. Jang JS, Lee A, Li J, Liyanage H, Yang Y, Guo L, Asmann YW, Li PW, Erickson-Johnson M, Sakai Y, Sun Z, Jeon HS, Hwang H, et al. Common oncogene mutations and novel snd1-braf transcript fusion in lung adenocarcinoma from never smokers. Sci Rep. 2015; 5:9755.
55. Giacomini CP, Sun S, Varma S, Shain AH, Giacomini MM, Balagtas J, Sweeney RT, Lai E, Del Vecchio CA, Forster AD, Clarke N, Montgomery KD, Zhu S, et al. Breakpoint analysis of transcriptional and genomic profiles uncovers novel gene fusions spanning multiple human cancer types. PLoS Genet. 2013; 9:e1003464.
56. Dessars B, De Raeve LE, El Housni H, Debouck CJ, Sidon PJ, Morandini R, Roseeuw D, Ghanem GE, Vassart G, Heimann P. Chromosomal translocations as a mechanism of BRAF activation in two cases of large congenital melanocytic nevi. J Invest Dermatol. 2007; 127:1468–70.
57. Palanisamy N, Ateeq B, Kalyana-Sundaram S, Pflueger D, Ramnarayanan K, Shankar S, Han B, Cao Q, Cao X, Suleman K, Kumar-Sinha C, Dhanasekaran SM, Chen YB, et al. Rearrangements of the RAF kinase pathway in prostate cancer, gastric cancer and melanoma. Nat Med. 2010; 16:793–98.
58. Ciampi R, Knauf JA, Kerler R, Gandhi M, Zhu Z, Nikiforova MN, Rabes HM, Fagin JA, Nikiforov YE. Oncogenic AKAP9-BRAF fusion is a novel mechanism of MAPK pathway activation in thyroid cancer. J Clin Invest. 2005; 115:94–101.
59. Yde CW, Sehested A, Mateu-Regué À, Østrup O, Scheie D, Nysom K, Nielsen FC, Rossing M. A new NFIA:RAF1 fusion activating the MAPK pathway in pilocytic astrocytoma. Cancer Genet. 2016; 209:440–44.
60. Baca SC, Prandi D, Lawrence MS, Mosquera JM, Romanel A, Drier Y, Park K, Kitabayashi N, MacDonald TY, Ghandi M, Van Allen E, Kryukov GV, Sboner A, et al. Punctuated evolution of prostate cancer genomes. Cell. 2013; 153:666–77.
61. Tomlins SA, Laxman B, Dhanasekaran SM, Helgeson BE, Cao X, Morris DS, Menon A, Jing X, Cao Q, Han B, Yu J, Wang L, Montie JE, et al. Distinct classes of chromosomal rearrangements create oncogenic ETS gene fusions in prostate cancer. Nature. 2007; 448:595–99.
62. Tomlins SA, Rhodes DR, Perner S, Dhanasekaran SM, Mehra R, Sun XW, Varambally S, Cao X, Tchinda J, Kuefer R, Lee C, Montie JE, Shah RB, et al. Recurrent fusion of TMPRSS2 and ETS transcription factor genes in prostate cancer. Science. 2005; 310:644–48.
63. Zack TI, Schumacher SE, Carter SL, Cherniack AD, Saksena G, Tabak B, Lawrence MS, Zhsng CZ, Wala J, Mermel CH, Sougnez C, Gabriel SB, Hernandez B, et al. Pan-cancer patterns of somatic copy number alteration. Nat Genet. 2013; 45:1134–40.
64. Tomlins SA, Rubin MA, Chinnaiyan AM. Integrative biology of prostate cancer progression. Annu Rev Pathol. 2006; 1:243–71.
65. Helgeson BE, Tomlins SA, Shah N, Laxman B, Cao Q, Prensner JR, Cao X, Singla N, Montie JE, Varambally S, Mehra R, Chinnaiyan AM. Characterization of TMPRSS2:ETV5 and SLC45A3:ETV5 gene fusions in prostate cancer. Cancer Res. 2008; 68:73–80.
66. Attard G, Clark J, Ambroisine L, Mills IG, Fisher G, Flohr P, Reid A, Edwards S, Kovacs G, Berney D, Foster C, Massie CE, Fletcher A, et al, and Transatlantic Prostate Group. Heterogeneity and clinical significance of ETV1 translocations in human prostate cancer. Br J Cancer. 2008; 99:314–20.
67. Han B, Mehra R, Dhanasekaran SM, Yu J, Menon A, Lonigro RJ, Wang X, Gong Y, Wang L, Shankar S, Laxman B, Shah RB, Varambally S, et al. A fluorescence *in situ* hybridization screen for E26 transformation-specific aberrations: identification of DDX5-ETV4 fusion protein in prostate cancer. Cancer Res. 2008; 68:7629–37.

68. Hermans KG, Bressers AA, van der Korput HA, Dits NF, Jenster G, Trapman J. Two unique novel prostate-specific and androgen-regulated fusion partners of ETV4 in prostate cancer. Cancer Res. 2008; 68:3094–98.

69. Paulo P, Barros-Silva JD, Ribeiro FR, Ramalho-Carvalho J, Jerónimo C, Henrique R, Lind GE, Skotheim RI, Lothe RA, Teixeira MR. FLI1 is a novel ETS transcription factor involved in gene fusions in prostate cancer. Genes Chromosomes Cancer. 2012; 51:240–49.

70. Northcott PA, Shih DJ, Peacock J, Garzia L, Morrissy AS, Zichner T, Stütz AM, Korshunov A, Reimand J, Schumacher SE, Beroukhim R, Ellison DW, Marshall CR, et al. Subgroup-specific structural variation across 1,000 medulloblastoma genomes. Nature. 2012; 488:49–56.

71. Geurts JM, Schoenmakers EF, Röijer E, Aström AK, Stenman G, van de Ven WJ. Identification of NFIB as recurrent translocation partner gene of HMGIC in pleomorphic adenomas. Oncogene. 1998; 16:865–72.

72. Brayer KJ, Frerich CA, Kang H, Ness SA. Recurrent fusions in myb and mybl1 define a common, transcription factor-driven oncogenic pathway in salivary gland adenoid cystic carcinoma. Cancer Discov. 2016; 6:176–87.

73. Fehr A, Kovács A, Löning T, Frierson H Jr, van den Oord J, Stenman G. The MYB-NFIB gene fusion-a novel genetic link between adenoid cystic carcinoma and dermal cylindroma. J Pathol. 2011; 224:322–27.

74. Mitani Y, Rao PH, Futreal PA, Roberts DB, Stephens PJ, Zhao YJ, Zhang L, Mitani M, Weber RS, Lippman SM, Caulin C, El-Naggar AK. Novel chromosomal rearrangements and break points at the t(6;9) in salivary adenoid cystic carcinoma: association with MYB-NFIB chimeric fusion, MYB expression, and clinical outcome. Clin Cancer Res. 2011; 17:7003–14.

75. Persson M, Andrén Y, Mark J, Horlings HM, Persson F, Stenman G. Recurrent fusion of MYB and NFIB transcription factor genes in carcinomas of the breast and head and neck. Proc Natl Acad Sci USA. 2009; 106:18740–44.

76. Persson M, Andrén Y, Moskaluk CA, Frierson HF Jr, Cooke SL, Futreal PA, Kling T, Nelander S, Nordkvist A, Persson F, Stenman G. Clinically significant copy number alterations and complex rearrangements of MYB and NFIB in head and neck adenoid cystic carcinoma. Genes Chromosomes Cancer. 2012; 51:805–17.

77. Bandopadhayay P, Ramkissoon LA, Jain P, Bergthold G, Wala J, Zeid R, Schumacher SE, Urbanski L, O'Rourke R, Gibson WJ, Pelton K, Ramkissoon SH, Han HJ, et al. MYB-QKI rearrangements in angiocentric glioma drive tumorigenicity through a tripartite mechanism. Nat Genet. 2016; 48:273–82.

78. Seshagiri S, Stawiski EW, Durinck S, Modrusan Z, Storm EE, Conboy CB, Chaudhuri S, Guan Y, Janakiraman V, Jaiswal BS, Guillory J, Ha C, Dijkgraaf GJ, et al. Recurrent R-spondin fusions in colon cancer. Nature. 2012; 488:660–64.

79. Salzman J, Marinelli RJ, Wang PL, Green AE, Nielsen JS, Nelson BH, Drescher CW, Brown PO. ESRRA-C11orf20 is a recurrent gene fusion in serous ovarian carcinoma. PLoS Biol. 2011; 9:e1001156.

80. Shin DH, Lee D, Hong DW, Hong SH, Hwang JA, Lee BI, You HJ, Lee GK, Kim IH, Lee YS, Han JY. Oncogenic function and clinical implications of SLC3A2-NRG1 fusion in invasive mucinous adenocarcinoma of the lung. Oncotarget. 2016; 7:69450–65. https://doi.org/10.18632/oncotarget.11913.

81. Cheng Y, Wang Y, Li J, Chang I, Wang CY. A novel read-through transcript JMJD7-PLA2G4B regulates head and neck squamous cell carcinoma cell proliferation and survival. Oncotarget. 2017; 8:1972–82. https://doi.org/10.18632/oncotarget.14081.

82. Graham RP, Jin L, Knutson DL, Kloft-Nelson SM, Greipp PT, Waldburger N, Roessler S, Longerich T, Roberts LR, Oliveira AM, Halling KC, Schirmacher P, Torbenson MS. DNAJB1-PRKACA is specific for fibrolamellar carcinoma. Mod Pathol. 2015; 28:822–29.

83. French CA, Miyoshi I, Aster JC, Kubonishi I, Kroll TG, Dal Cin P, Vargas SO, Perez-Atayde AR, Fletcher JA. BRD4 bromodomain gene rearrangement in aggressive carcinoma with translocation t(15;19). Am J Pathol. 2001; 159:1987–92.

84. Linehan WM, Spellman PT, Ricketts CJ, Creighton CJ, Fei SS, Davis C, Wheeler DA, Murray BA, Schmidt L, Vocke CD, Peto M, Al Mamun AA, Shinbrot E, et al, and Cancer Genome Atlas Research Network. Comprehensive molecular characterization of papillary renal-cell carcinoma. N Engl J Med. 2016; 374:135–45.

85. Davis IJ, Hsi BL, Arroyo JD, Vargas SO, Yeh YA, Motyckova G, Valencia P, Perez-Atayde AR, Argani P, Ladanyi M, Fletcher JA, Fisher DE. Cloning of an Alpha-TFEB fusion in renal tumors harboring the t(6;11)(p21;q13) chromosome translocation. Proc Natl Acad Sci USA. 2003; 100:6051–56.

86. Xie H, Rachakonda PS, Heidenreich B, Nagore E, Sucker A, Hemminki K, Schadendorf D, Kumar R. Mapping of deletion breakpoints at the CDKN2A locus in melanoma: detection of MTAP-ANRIL fusion transcripts. Oncotarget. 2016; 7:16490–504. https://doi.org/10.18632/oncotarget.7503.

87. Yao F, Kausalya JP, Sia YY, Teo AS, Lee WH, Ong AG, Zhang Z, Tan JH, Li G, Bertrand D, Liu X, Poh HM, Guan P, et al. Recurrent fusion genes in gastric cancer: Cldn18-arhgap26 induces loss of epithelial integrity. Cell Rep. 2015; 12:272–85.

88. Kalvala A, Gao L, Aguila B, Dotts K, Rahman M, Nana-Sinkam SP, Zhou X, Wang QE, Amann J, Otterson GA, Villalona-Calero MA, Duan W. Rad51C-ATXN7 fusion gene expression in colorectal tumors. Mol Cancer. 2016; 15:47.

89. Tsuda M, Davis IJ, Argani P, Shukla N, McGill GG, Nagai M, Saito T, Laé M, Fisher DE, Ladanyi M. TFE3 fusions

activate MET signaling by transcriptional up-regulation, defining another class of tumors as candidates for therapeutic MET inhibition. Cancer Res. 2007; 67:919–29.

90. Li C, Cao J, Zhang N, Tu M, Xu F, Wei S, Chen X, Xu Y. Identification of rspo2 fusion mutations and target therapy using a porcupine inhibitor. Sci Rep. 2018; 8:14244.
91. Han T, Schatoff EM, Murphy C, Zafra MP, Wilkinson JE, Elemento O, Dow LE. R-Spondin chromosome rearrangements drive Wnt-dependent tumour initiation and maintenance in the intestine. Nat Commun. 2017; 8:15945.
92. Picco G, Petti C, Centonze A, Torchiaro E, Crisafulli G, Novara L, Acquaviva A, Bardelli A, Medico E. Loss of AXIN1 drives acquired resistance to WNT pathway blockade in colorectal cancer cells carrying RSPO3 fusions. EMBO Mol Med. 2017; 9:293–303.
93. Robinson DR, Kalyana-Sundaram S, Wu YM, Shankar S, Cao X, Ateeq B, Asangani IA, Iyer M, Maher CA, Grasso CS, Lonigro RJ, Quist M, Siddiqui J, et al. Functionally recurrent rearrangements of the MAST kinase and Notch gene families in breast cancer. Nat Med. 2011; 17:1646–51.
94. Blee AM, He Y, Yang Y, Ye Z, Yan Y, Pan Y, Ma T, Dugdale J, Kuehn E, Kohli M, Jimenez R, Chen Y, Xu W, et al. Tmprss2-erg controls luminal epithelial lineage and antiandrogen sensitivity in pten and tp53-mutated prostate cancer. Clin Cancer Res. 2018; 24:4551–65.
95. Bresler SC, Wood AC, Haglund EA, Courtright J, Belcastro LT, Plegaria JS, Cole K, Toporovskaya Y, Zhao H, Carpenter EL, Christensen JG, Maris JM, Lemmon MA, Mossé YP. Differential inhibitor sensitivity of anaplastic lymphoma kinase variants found in neuroblastoma. Sci Transl Med. 2011; 3:108ra114.
96. Choi YL, Soda M, Yamashita Y, Ueno T, Takashima J, Nakajima T, Yatabe Y, Takeuchi K, Hamada T, Haruta H, Ishikawa Y, Kimura H, Mitsudomi T, et al, and ALK Lung Cancer Study Group. EML4-ALK mutations in lung cancer that confer resistance to ALK inhibitors. N Engl J Med. 2010; 363:1734–39.
97. Christensen JG, Zou HY, Arango ME, Li Q, Lee JH, McDonnell SR, Yamazaki S, Alton GR, Mroczkowski B, Los G. Cytoreductive antitumor activity of PF-2341066, a novel inhibitor of anaplastic lymphoma kinase and c-Met, in experimental models of anaplastic large-cell lymphoma. Mol Cancer Ther. 2007; 6:3314–22.
98. Doebele RC, Pilling AB, Aisner DL, Kutateladze TG, Le AT, Weickhardt AJ, Kondo KL, Linderman DJ, Heasley LE, Franklin WA, Varella-Garcia M, Camidge DR. Mechanisms of resistance to crizotinib in patients with ALK gene rearranged non-small cell lung cancer. Clin Cancer Res. 2012; 18:1472–82.
99. Katayama R, Shaw AT, Khan TM, Mino-Kenudson M, Solomon BJ, Halmos B, Jessop NA, Wain JC, Yeo AT, Benes C, Drew L, Saeh JC, Crosby K, et al. Mechanisms of acquired crizotinib resistance in ALK-rearranged lung Cancers. Sci Transl Med. 2012; 4:120ra17.
100. Lu H, Villafane N, Dogruluk T, Grzeskowiak CL, Kong K, Tsang YH, Zagorodna O, Pantazi A, Yang L, Neill NJ, Kim YW, Creighton CJ, Verhaak RG, et al. Engineering and functional characterization of fusion genes identifies novel oncogenic drivers of cancer. Cancer Res. 2017; 77:3502–12.
101. Sasaki T, Okuda K, Zheng W, Butrynski J, Capelletti M, Wang L, Gray NS, Wilner K, Christensen JG, Demetri G, Shapiro GI, Rodig SJ, Eck MJ, Jänne PA. The neuroblastoma-associated F1174L ALK mutation causes resistance to an ALK kinase inhibitor in ALK-translocated cancers. Cancer Res. 2010; 70:10038–43.
102. Zhang S, Wang F, Keats J, Zhu X, Ning Y, Wardwell SD, Moran L, Mohemmad QK, Anjum R, Wang Y, Narasimhan NI, Dalgarno D, Shakespeare WC, et al. Crizotinib-resistant mutants of EML4-ALK identified through an accelerated mutagenesis screen. Chem Biol Drug Des. 2011; 78:999–1005.
103. Golding B, Luu A, Jones R, Viloria-Petit AM. The function and therapeutic targeting of anaplastic lymphoma kinase (ALK) in non-small cell lung cancer (NSCLC). Mol Cancer. 2018; 17:52.
104. Lovly CM, Heuckmann JM, de Stanchina E, Chen H, Thomas RK, Liang C, Pao W. Insights into ALK-driven cancers revealed through development of novel ALK tyrosine kinase inhibitors. Cancer Res. 2011; 71:4920–31.
105. Mologni L, Ceccon M, Pirola A, Chiriano G, Piazza R, Scapozza L, Gambacorti-Passerini C. NPM/ALK mutants resistant to ASP3026 display variable sensitivity to alternative ALK inhibitors but succumb to the novel compound PF-06463922. Oncotarget. 2015; 6:5720–34. https://doi.org/10.18632/oncotarget.3122.
106. Sakamoto H, Tsukaguchi T, Hiroshima S, Kodama T, Kobayashi T, Fukami TA, Oikawa N, Tsukuda T, Ishii N, Aoki Y. CH5424802, a selective ALK inhibitor capable of blocking the resistant gatekeeper mutant. Cancer Cell. 2011; 19:679–90.
107. Zhu VW, Schrock AB, Bosemani T, Benn BS, Ali SM, Ou SI. Dramatic response to alectinib in a lung cancer patient with a novel VKORC1L1-ALK fusion and an acquired ALK T1151K mutation. Lung Cancer (Auckl). 2018; 9:111–16.
108. Ardini E, Galvani A. Alk inhibitors, a pharmaceutical perspective. Front Oncol. 2012; 2:17.
109. Friboulet L, Li N, Katayama R, Lee CC, Gainor JF, Crystal AS, Michellys PY, Awad MM, Yanagitani N, Kim S, Pferdekamper AC, Li J, Kasibhatla S, et al. The ALK inhibitor ceritinib overcomes crizotinib resistance in non-small cell lung cancer. Cancer Discov. 2014; 4:662–73.
110. Katayama R, Khan TM, Benes C, Lifshits E, Ebi H, Rivera VM, Shakespeare WC, Iafrate AJ, Engelman JA, Shaw AT. Therapeutic strategies to overcome crizotinib resistance in non-small cell lung cancers harboring the fusion oncogene EML4-ALK. Proc Natl Acad Sci USA. 2011; 108:7535–40.

111. Koivunen JP, Mermel C, Zejnullahu K, Murphy C, Lifshits E, Holmes AJ, Choi HG, Kim J, Chiang D, Thomas R, Lee J, Richards WG, Sugarbaker DJ, et al. EML4-ALK fusion gene and efficacy of an ALK kinase inhibitor in lung cancer. Clin Cancer Res. 2008; 14:4275–83.

112. Amatu A, Somaschini A, Cerea G, Bosotti R, Valtorta E, Buonandi P, Marrapese G, Veronese S, Luo D, Hornby Z, Multani P, Murphy D, Shoemaker R, et al. Novel CAD-ALK gene rearrangement is drugable by entrectinib in colorectal cancer. Br J Cancer. 2015; 113:1730–34.

113. Lee J, Kim HC, Hong JY, Wang K, Kim SY, Jang J, Kim ST, Park JO, Lim HY, Kang WK, Park YS, Lee J, Lee WY, et al. Detection of novel and potentially actionable anaplastic lymphoma kinase (ALK) rearrangement in colorectal adenocarcinoma by immunohistochemistry screening. Oncotarget. 2015; 6:24320–32. https://doi.org/10.18632/oncotarget.4462.

114. Hong S, Chen N, Fang W, Zhan J, Liu Q, Kang S, He X, Liu L, Zhou T, Huang J, Chen Y, Qin T, Zhang Y, et al. Upregulation of PD-L1 by EML4-ALK fusion protein mediates the immune escape in ALK positive NSCLC: implication for optional anti-PD-1/PD-L1 immune therapy for ALK-TKIs sensitive and resistant NSCLC patients. OncoImmunology. 2015; 5:e1094598.

115. André F, Bachelot T, Campone M, Dalenc F, Perez-Garcia JM, Hurvitz SA, Turner N, Rugo H, Smith JW, Deudon S, Shi M, Zhang Y, Kay A, et al. Targeting FGFR with dovitinib (TKI258): preclinical and clinical data in breast cancer. Clin Cancer Res. 2013; 19:3693–702.

116. Byron SA, Chen H, Wortmann A, Loch D, Gartside MG, Dehkhoda F, Blais SP, Neubert TA, Mohammadi M, Pollock PM. The N550K/H mutations in FGFR2 confer differential resistance to PD173074, dovitinib, and ponatinib ATP-competitive inhibitors. Neoplasia. 2013; 15:975–88.

117. Konecny GE, Kolarova T, O'Brien NA, Winterhoff B, Yang G, Qi J, Qi Z, Venkatesan N, Ayala R, Luo T, Finn RS, Kristof J, Galderisi C, et al. Activity of the fibroblast growth factor receptor inhibitors dovitinib (TKI258) and NVP-BGJ398 in human endometrial cancer cells. Mol Cancer Ther. 2013; 12:632–42.

118. Lim SM, Kim HR, Shim HS, Soo RA, Cho BC. Role of FGF receptors as an emerging therapeutic target in lung squamous cell carcinoma. Future Oncol. 2013; 9:377–86.

119. Arai Y, Totoki Y, Hosoda F, Shirota T, Hama N, Nakamura H, Ojima H, Furuta K, Shimada K, Okusaka T, Kosuge T, Shibata T. Fibroblast growth factor receptor 2 tyrosine kinase fusions define a unique molecular subtype of cholangiocarcinoma. Hepatology. 2014; 59:1427–34.

120. Capelletti M, Dodge ME, Ercan D, Hammerman PS, Park SI, Kim J, Sasaki H, Jablons DM, Lipson D, Young L, Stephens PJ, Miller VA, Lindeman NI, et al. Identification of recurrent FGFR3-TACC3 fusion oncogenes from lung adenocarcinoma. Clin Cancer Res. 2014; 20:6551–58.

121. Guagnano V, Kauffmann A, Wöhrle S, Stamm C, Ito M, Barys L, Pornon A, Yao Y, Li F, Zhang Y, Chen Z, Wilson CJ, Bordas V, et al. FGFR genetic alterations predict for sensitivity to NVP-BGJ398, a selective pan-FGFR inhibitor. Cancer Discov. 2012; 2:1118–33.

122. Liao RG, Jung J, Tchaicha J, Wilkerson MD, Sivachenko A, Beauchamp EM, Liu Q, Pugh TJ, Pedamallu CS, Hayes DN, Gray NS, Getz G, Wong KK, et al. Inhibitor-sensitive FGFR2 and FGFR3 mutations in lung squamous cell carcinoma. Cancer Res. 2013; 73:5195–205.

123. Wang Y, Ding X, Wang S, Moser CD, Shaleh HM, Mohamed EA, Chaiteerakij R, Allotey LK, Chen G, Miyabe K, McNulty MS, Ndzengue A, Barr Fritcher EG, et al. Antitumor effect of FGFR inhibitors on a novel cholangiocarcinoma patient derived xenograft mouse model endogenously expressing an FGFR2-CCDC6 fusion protein. Cancer Lett. 2016; 380:163–73.

124. Yuan L, Liu ZH, Lin ZR, Xu LH, Zhong Q, Zeng MS. Recurrent FGFR3-TACC3 fusion gene in nasopharyngeal carcinoma. Cancer Biol Ther. 2014; 15:1613–21.

125. Liu H, Murphy CJ, Karreth FA, Emdal KB, White FM, Elemento O, Toker A, Wulf GM, Cantley LC. Identifying and targeting sporadic oncogenic genetic aberrations in mouse models of triple-negative breast cancer. Cancer Discov. 2018; 8:354–69.

126. Nakanishi Y, Akiyama N, Tsukaguchi T, Fujii T, Satoh Y, Ishii N, Aoki M. Mechanism of oncogenic signal activation by the novel fusion kinase fgfr3-baiap2l1. Mol Cancer Ther. 2015; 14:704–12.

127. Konicek BW, Capen AR, Credille KM, Ebert PJ, Falcon BL, Heady GL, Patel BK, Peek VL, Stephens JR, Stewart JA, Stout SL, Timm DE, Um SL, et al. Merestinib (LY2801653) inhibits neurotrophic receptor kinase (NTRK) and suppresses growth of NTRK fusion bearing tumors. Oncotarget. 2018; 9:13796–806. https://doi.org/10.18632/oncotarget.24488.

128. Jain P, Silva A, Han HJ, Lang SS, Zhu Y, Boucher K, Smith TE, Vakil A, Diviney P, Choudhari N, Raman P, Busch CM, Delaney T, et al. Overcoming resistance to single-agent therapy for oncogenic BRAF gene fusions via combinatorial targeting of MAPK and PI3K/mTOR signaling pathways. Oncotarget. 2017; 8:84697–713. https://doi.org/10.18632/oncotarget.20949.

129. Sievert AJ, Lang SS, Boucher KL, Madsen PJ, Slaunwhite E, Choudhari N, Kellet M, Storm PB, Resnick AC. Paradoxical activation and RAF inhibitor resistance of BRAF protein kinase fusions characterizing pediatric astrocytomas. Proc Natl Acad Sci USA. 2013; 110:5957–62.

130. Chmielecki J, Hutchinson KE, Frampton GM, Chalmers ZR, Johnson A, Shi C, Elvin J, Ali SM, Ross JS, Basturk O, Balasubramanian S, Lipson D, Yelensky R, et al. Comprehensive genomic profiling of pancreatic acinar cell carcinomas identifies recurrent RAF fusions and frequent inactivation of DNA repair genes. Cancer Discov. 2014; 4:1398–405.

131. Ross JS, Wang K, Chmielecki J, Gay L, Johnson A, Chudnovsky J, Yelensky R, Lipson D, Ali SM, Elvin JA, Vergilio JA, Roels S, Miller VA, et al. The distribution of

BRAF gene fusions in solid tumors and response to targeted therapy. Int J Cancer. 2016; 138:881–90.

132. Turner JA, Bemis JG, Bagby SM, Capasso A, Yacob BW, Chimed TS, Van Gulick R, Lee H, Tobin R, Tentler JJ, Pitts T, McCarter M, Robinson WA, Couts KL. BRAF fusions identified in melanomas have variable treatment responses and phenotypes. Oncogene. 2019; 38:1296–308. https://doi.org/10.1038/s41388-018-0514-7.

133. Matissek KJ, Onozato ML, Sun S, Zheng Z, Schultz A, Lee J, Patel K, Jerevall PL, Saladi SV, Macleay A, Tavallai M, Badovinac-Crnjevic T, Barrios C, et al. Expressed gene fusions as frequent drivers of poor outcomes in hormone receptor-positive breast cancer. Cancer Discov. 2018; 8:336–53.

134. Paratala BS, Chung JH, Williams CB, Yilmazel B, Petrosky W, Williams K, Schrock AB, Gay LM, Lee E, Dolfi SC, Pham K, Lin S, Yao M, et al. RET rearrangements are actionable alterations in breast cancer. Nat Commun. 2018; 9:4821.

135. Matsubara D, Kanai Y, Ishikawa S, Ohara S, Yoshimoto T, Sakatani T, Oguni S, Tamura T, Kataoka H, Endo S, Murakami Y, Aburatani H, Fukayama M, Niki T. Identification of CCDC6-RET fusion in the human lung adenocarcinoma cell line, LC-2/ad. J Thorac Oncol. 2012; 7:1872–76.

136. Nelson-Taylor SK, Le AT, Yoo M, Schubert L, Mishall KM, Doak A, Varella-Garcia M, Tan AC, Doebele RC. Resistance to ret-inhibition in ret-rearranged nsclc is mediated by reactivation of ras/mapk signaling. Mol Cancer Ther. 2017; 16:1623–33.

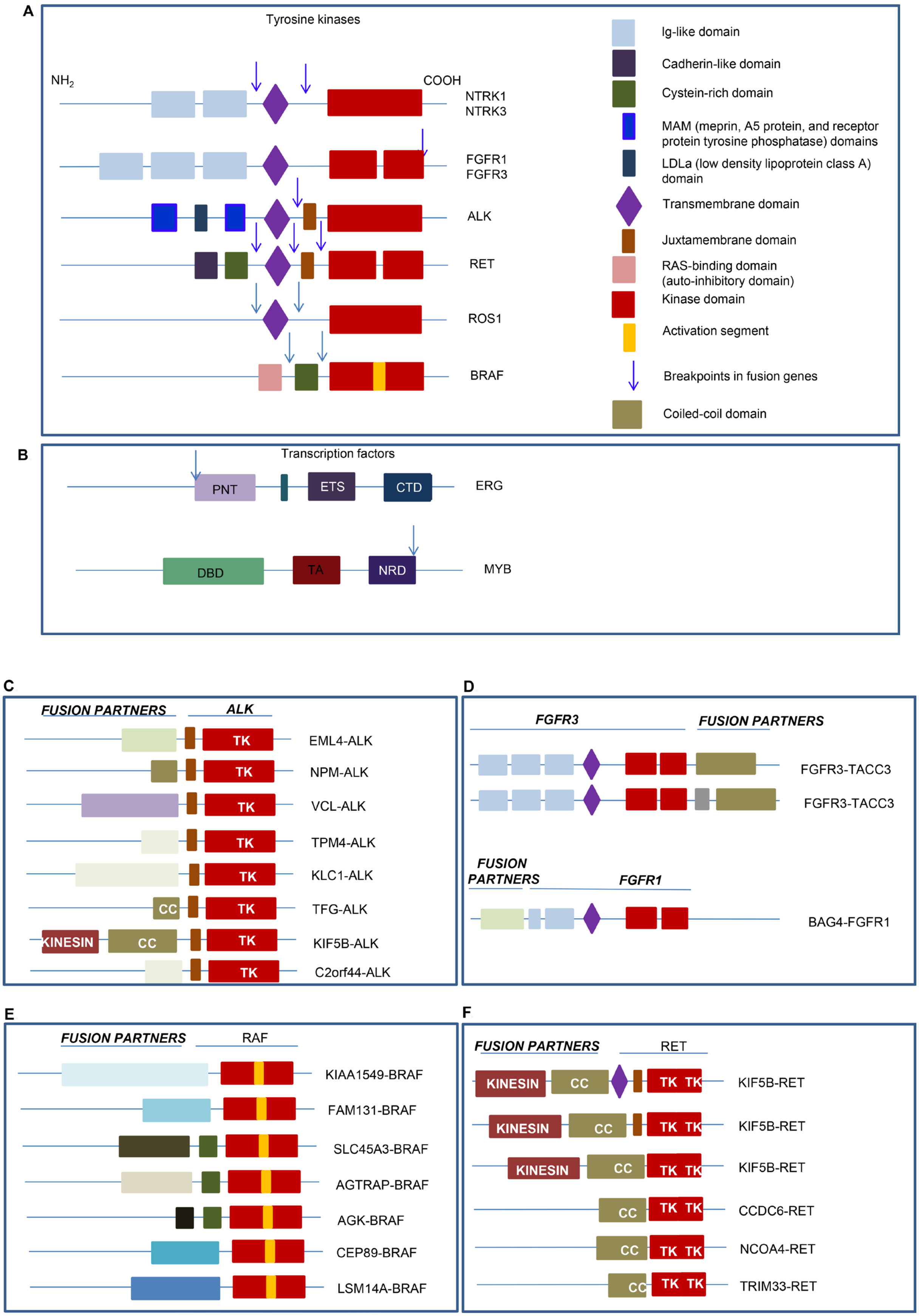
A
Tyrosine kinases
NH2
COOH
NTRK1
NTRK3
FGFR1
FGFR3
ALK
RET
ROS1
BRAF
Ig-like domain
Cadherin-like domain
Cystein-rich domain
MAM (meprin, A5 protein, and receptor protein tyrosine phosphatase) domains
LDLa (low density lipoprotein class A) domain
Transmembrane domain
Juxtamembrane domain
RAS-binding domain (auto-inhibitory domain)
Kinase domain
Activation segment
Breakpoints in fusion genes
Coiled-coil domain
B
Transcription factors
PNT
ETS
CTD
ERG
DBD
TA
NRD
MYB
C
FUSION PARTNERS
ALK
TK
EML4-ALK
NPM-ALK
VCL-ALK
TPM4-ALK
KLC1-ALK
CC
TFG-ALK
KINESIN
KIF5B-ALK
C2orf44-ALK
D
FGFR3
FUSION PARTNERS
FGFR3-TACC3
FGFR3-TACC3
FUSION PARTNERS
FGFR1
BAG4-FGFR1
E
FUSION PARTNERS
RAF
KIAA1549-BRAF
FAM131-BRAF
SLC45A3-BRAF
AGTRAP-BRAF
AGK-BRAF
CEP89-BRAF
LSM14A-BRAF
F
FUSION PARTNERS
RET
KIF5B-RET
KIF5B-RET
KIF5B-RET
CCDC6-RET
NCOA4-RET
TRIM33-RET

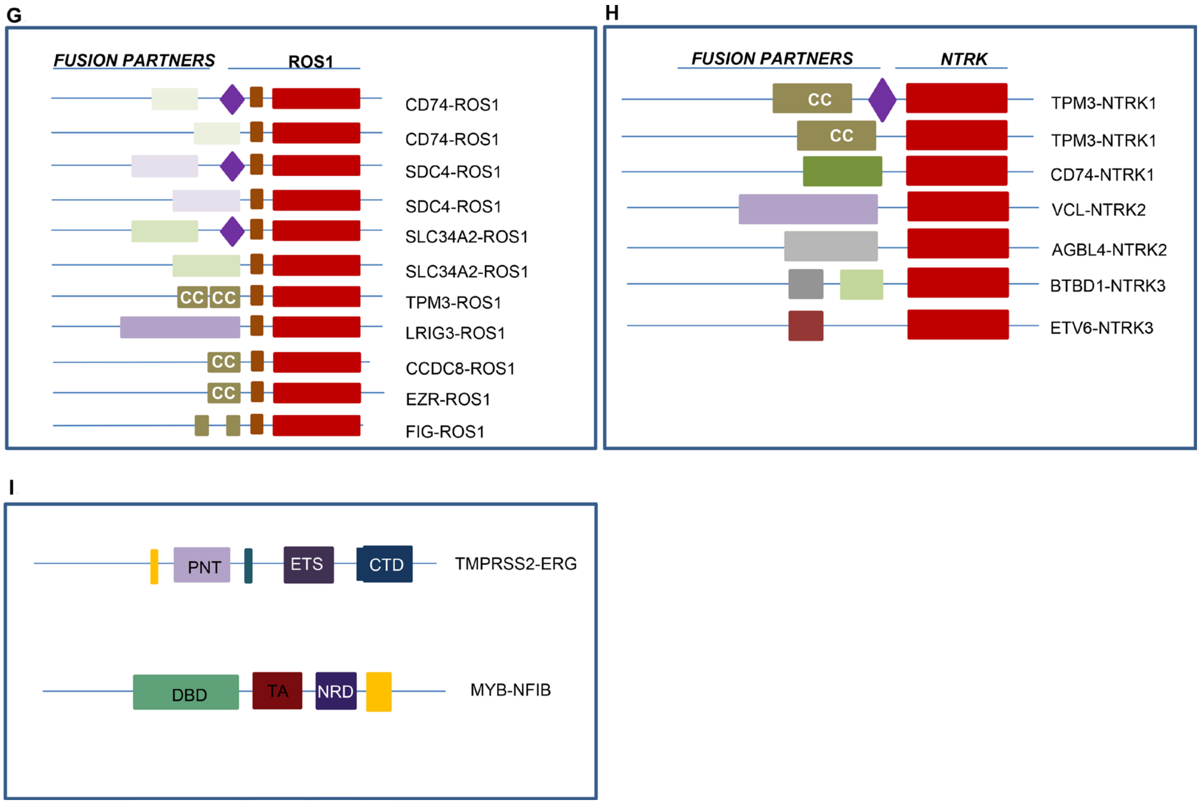

Supplementary Figure 1: Shematic representations of tyrosine kinases (TKs) and transcription factors (TFs), their domains their partners that involve to formation of fusion genes. Breakpoints of (**A**) TKs and (**B**) TFs. ERG protein consists PNT, pointed protein-protein interaction domain; AD, alternative domain; ETS domain that forms a helix-turn-helix DNA-binding domain; CTD, C-terminal transactivation domain. MYB protein consist three functional domains; helix-turn-helix (HTH)-type DNA binding domain (DBD) in N-terminal, trans-activation domain (TAD) and negative regulatory domain (NRD) in C-terminal. (**C**) Some fusion partners of ALK, (**D**) FGFR, (**E**) RAF, (**F**) RET, (**G**) ROS1, (**H**) NTRK, (**I**) ERG and MYB.
