# Supplementary material for: Molecular mechanisms and pathobiology of oncogenic fusion transcripts in epithelial tumors
Source: Oncotarget. 2019 Mar 12;10(21):2095–111. doi: 10.18632/oncotarget.26777 (PMC6459343; doi:10.18632/oncotarget.26777)
Supplement: Supplementary file 2 [file oncotarget-10-2095-s002.docx]

**Supplementary Table 1.** Examples of oncogenic fusion genes of receptor tyrosine kinases in epithelial tumors.

| **Fusion gene** | **Tumor type** | **Type of rearrangements** | **Localization** | **Fusion gene domains** | **Refs** |
| --- | --- | --- | --- | --- | --- |
| ***ALK partners*** | | | | | |
| *EML4*  *TFG*  *KIF5B*  *KLC1*  *C2orf44*  *NPM*  *VCL*  *TPM4*  *TPM3*  *STRN* | NSCLC (1-11%), BRCA (2.5%), CRC (2.4%), SNMBC  NSCLC, SNMBC  NSCLC  CRC  ALCL  RCC  Esophageal carcinoma  Adult and pediatric RCC  Advanced metastatic CRC  PTC (9%), ATC (4%) | INV  TR  TR  TR  TD, RT  DEL  TR  TR  TR  Chromotripsis, TR | PM  Cytoplasm  Cytoplasm  Cytoplasm  Cytoplasm  Cytoplasm  Cytoplasm  Cytoplasm  Cytoplasm  Cytoplasm  Cytoplasm | CC-JM-TK, CC-TM-JM-TK  CC-WDRR_3_-JM-TK  PB1-CC-JM-TK  KINESIN-CC-JM-TK  N-JM-TK  N-JM-TK  CC-JM-TK  AB-TK  CC-JM_TK  CC-JM-TK  CB-CC-JM-TK | [1-4]  [5]  [6]  [7]  [4]  [8]  [9]  [10]  [11]  [12,13] |
| ***FGFR3 partners*** | | | | | |
| *TACC3* | LUSC (1.1%), BLCA (2-10%), LGG (0.4%),  GBM (1.2-8.3%), LUAD, thyroid, oral, HNSCC | TD, RT | PM | Ig-Ig-Ig-TM-TK-TK-CC (GBM, HNSCC, BLCA, LUAD, LUSC), Ig-Ig-Ig-TM-TK-CC (lung) | [14-17] |
| *BAIAP2L1* | NSCC, cervical, BLCA | TR | PM | Ig-Ig-Ig-TM-TK-BAR | [15,17] |
| ***FGFR2*** | | | | | |
| *CCDC6*  *OFD1*  *BICC1*  *KIAA1967*  *AFF3*  *SLC45A3* | BR  Thyroid cancer  Cholangiocarcinoma  Cholangiocarcinoma  *BR*  Prostate | INTR  TR  INTR  TR  TR  TR | PM  PM  PM  PM  PM  PM | Ig-Ig-Ig-TM-TK-TK-CC  Ig-Ig-Ig-TM-TK-TK-LisH  Ig-Ig-Ig-TM-TK-TK-SAM  Ig-Ig-Ig-TM-TK-TK-CCAR2  Ig-Ig-Ig-TM-TK-TK-AFF3  Ig-Ig-Ig-TM-TK-TK-MFS | [17]  [17]  [17]  [17]  [17,18]  [17] |
| ***FGFR1*** | | | | | |
| BAG4  ERLIN2 | LUSC  BR | INTR  INTR | PM  PM | BAG4-Ig-TM-TK-TK  SPFH-Ig-Ig-Ig-TM-TK-TK | [17]  [17] |
| ***RET*** | | | | | |
| *KIF5B*  *CCDC6*  *NCOA4*  *KTN1*  *TRIM33* | NSCLC (1-2.8%)  PTC, NSCLC, Colon adenocarcinoma  PTC, NSCLC  PTC (childhood)  PTC^##^, LUAD | INV  INV  INV  TR  TR | Cytoplasm  PM  Cytoplasm  Cytoplasm  Cytoplasm | KINESIN-CC-TK; KINESIN-CC-JM-TK  KINESIN-CC-TM-JM-TK  CC-TK  CC-TK  TM-CC-TK  CC-TK | [4,19-29],  [20,30,31]  [20,32]  [33]  [31] |
| ***ROS1*** | | | | | |
| *CD74*  *EZR*  *SLC34A2*  *TPM3*  *SDC4*  *LRIG3*  *GOPC (FIG)*  *SHTN1*  *KDELR2*  *CCDC6*  *MSN*  *PPFIBP1*  *ZCCHC8*  *PWWP2A* | NSCLC(2.5%), GBM  NSCLC  NSCLC, gastric cancer  NSCLC, Spitz nevus**  NSCLC  NSCLC  GBM, NSCLC, BTR, cholangiocarcinoma  Spitz nevus (25.3%), NSCLC-never smokers  LUAD  LUAD  LUAD  Spitz nevus  Spitz nevus**,  Atypical Spitz tumor | TR  PAR INV  TR  TR  TR  TR  DEL  TR  TR  TR  TR  TR  TR  TR | PM  Cytoplasm  PM  Cytoplasm  PM  PM  Golgi apparatus  Cytoplasm  Cytoplasm  PM  Cytoplasm  PM  PM  Cytoplasm  Cytoplasm | TM-EC-TM-TK  FERM-CCp-TK  TM_(1-5)_-EC-TM-TK  CC-CC-TK  EC-TM-TK  TM-TK?  CC-CC-TK (L)  CC-CC-LZ-TK (S)  CC-CC-CC-TK  TM-TM-TM-TK  CC-CC-TK  FERM-EC-TM-TK  CC-CC-CC-CC-TK  CC-TK  N-TK | [24,34,35]  [36]  [24,37]  [24,38]  [39-41]  [42]  [40,41,43]  [38,44]  [24]  [24]  [24]  [38]  [38]  [38] |
| ***NTRK3*** | | | | | |
| *ETV6* | SBC, SGC, SBRC, NBS, HGG, Colon | TR | Cytoplasm | PPPID-TK | [19,25,26,28,29,45-48] |
| ***NTRK2*** | | | | | |
| *NACC2*  *QKI* | PA  PA | INV  TR | Cytoplasm  Cytoplasm | QUA1*-TK | [49]  [49] |
| ***NTRK1*** | | | | | |
| *TPM3*  *CD74*  *MPRIP* | PTC, NBS-HGG (40%)  LUAD  LUAD | INV  TR  TR | Cytoplasm  PM  Cytoplasm | TRMD-TK  TM-OM-TM-TK  CC-CC-CC-TK | [50]  [51]  [51] |

Abbreviations: ^b^Deletion of JM inhibitory domain; *QUA1 is oligomerization domain; **Benign tumor; ^#^Also arises through chromoplexy; ^##^*TRIM33-RET, AKAP9-BRAF* have been reported in radiation-associated PTC; AS/RT, aberrant splicing/read through; TD, tandem duplication; TR, translocation; INTR, intrachromosomal translocation; INV, inversion; PAR INV, paracentric inversion; ADG, adult diffuse glioma; LUAD, lung adenocarcinoma; SCLC, squamous cell lung cancer; NSCLC, non–small cell lung cancer; SNMBC; small non-mucinous bronchio-alveolar carcinoma; HCC, hepatocellular carcinoma; CRC, colorectal cancer; RCC, renal cell cancer; GA, gastric cancer; SCM, skin cutaneous melanoma; ALCL, anaplastic large-cell lymphoma; BLCA, bladder cancer; BRCA, breast cancer; BACC, breast adenoid cystic carcinoma; SBA, secretory breast cancer; GBM, glioblastoma; OV, ovarian cancer; LG, low-grade; HG, high-grade; SNMBC, small non-mucinous bronchio-alveolar carcinoma; H&NACC, head and neck adenoid cystic carcinoma; SGC, salivary gland carcinoma; UCEC, uterine cervix cancer; PA, pilocytic astrocytoma; PCa, prostate cancer; ATC, anaplastic thyroid cancer; PTC, papillary thyroid cancer; BTR, biliary tract carcinoma. HTHDB, helix-turn-helix (HTH) DNA-binding motifs; TA, transactivation domain; NRD, negative regulatory domain; PK, protein serine/threonine kinase; PM, plasma membrane; CR, cysteine-rich domain; BRM, bromodomain; OM, oligomerization domain.
