# Supplementary material for: Molecular mechanisms and pathobiology of oncogenic fusion transcripts in epithelial tumors
Source: Oncotarget. 2019 Mar 12;10(21):2095–111. doi: 10.18632/oncotarget.26777 (PMC6459343; doi:10.18632/oncotarget.26777)
Supplement: Supplementary file 3 [file oncotarget-10-2095-s003.docx]

**Supplementary Table 5: Examples of oncogenic fusions that sensitive or resistant to targeted therapeutics agents**

| **Fusion Genes** | **Inhibitors** | **Resistance** | **Sensitive** | **Reference** |
| --- | --- | --- | --- | --- |
| *TFE3* | PHA665752  *MET-inhibitor* |  | ASPL-TFE3 expressing FU-UR-1 renal carcinoma cell | [89] |
| *RSPO* | CGX1321  *Porcupine- inhibitor* | Tumor cells without RSPO2 fusion | EIF3E(e1)-RSPO2(e2)  EMC2(e1)-RSPO2(e2)  HNF4G(e1)-RSPO2(e2)  PVT1(e1)-RSPO2(e2)  HNF4G(e1)-RSPO2(e3) gastric and colon cancer derived cells in PDX mouse model | [90] |
|  | LGK974  *Porcupin-inhibitor*  *(inhibit Wnt/β-catenin)* | Long term treatment with LGK974 led to developing resistance with carrying two frameshift deletion in Wnt pathway inhibitor AXIN1 | RTPRK(e1)-RSPO3(e2)  PTPRK(e13)-RSPO3(e2) in VACO6 cell lines,  EIF3E(e1)-RSPO2(e2)  PTPRK(e1)-RSPO3(e2) transgenic mice | [91,92] |
| *NOTCH* | DAPT^#^  *γ-secretase-inhibitor* | SEC22B(e1)-NOTCH2(e27)  SEC16A(e1)-NOTCH(e27) expressing HCC1187 cells (NOTCH fusions that encode NICD independent of the γ-secretase cleavage site),  Truncated NOTCH expressed HCC1559 cells | SEC16A(e2)-NOTCH1 (e28) expressing HCC2218 cells and mice xenograft model (NOTCH fusion that retain the γ-secretase cleavage site in ER-negative breast cancer) | [93] |
|  | Enzalutamide  *Antiandrogen* | PTEN and TP53 deleted or mutated, but TMPRSS2-ERG fusion negative cells are antiandrogen resistant, but CDK4/6 inhibitor sensitive | TMPRSS2(e1)-ERG(e4) positive/PTEN and TP53 deleted or mutated VCaP cells are antiandrogen sensitive but CDK4/6 inhibitor resistant) in vivo model | [94] |
| *ALK* | Crizotinib  (PF-02341066)  *ALK & ROS1& MET-inhibitor* | *CD74-ROS1^G2032R^*  *EML4-ALK*^L1196M,^ ^L1198P,^ ^G1202R,^ ^D1203N,^ ^S1206Y,^ ^1151Tins^  ALK^L1152R, G1296A, C1156Y^,  *ALK*, *EML4-ALK* or *KIT* amplified lung cancer,  EML4-ALK ^F1174L^, ALK^F1174L^ neuroblastoma  RANBP2-ALK^F1174L^ IMT  NPM-ALK ^G1128S, C1156F, I1171N/T, F1174I, N1178H, E1210K, C1156F/D1230N^ in Ba/F3 cell model  ALK^G1269A, S1206Y, V1180L, G1202R, C1156Y,1151ins, F1174C/L, L1198P, L1152R/P, I1171N/T, D1203N^  EML4(e13)-ALK(e20)/C1156Y in Ba/F3 cells  EML4(e13)-ALK(e20)/L1196M in Ba/F3 cells, ALK^F1174L^ expressing SY5Y neuroblastoma cells, HepG2 hepatocellular carcinoma cells, KRAS ^G12C^ expressing H2020 lung cancer cells, SLC34A2-ROS expressing HCC78 lung cancer cells | ALK^R1275Q^ , NPM1-ALK neuroblastoma  NPM-ALK ^C1156F, F1174I, N1178H, E1210K^ in Ba/F3 cell model  EML4-ALK expressing Ba/F3 cells  EML4(e13)-ALK(e20) I cell n H3122 lung cancer | [34,95-104] |
|  | Alectinib^#^^  CH5424802  (RO542802802)  *ALK-inhibitor* | *EML4-ALK* ^G1202R^, ^1151Tins, V1180L, G1202R^  lung cancer  NPM-ALK^G1128S, C1156F, I1171N/T, E1210K, C1156F/D1230N^ in Ba/F3 cells  ALK^I1171T/N^ in patients  ALK^V1180L, G1202R^ in Ba/F3 models  MET amplification, overexpression of neuregulin-1 | EML4-ALK, EML4-ALK^L1196M, S1206Y^, ALK^L1196M^ NSCLC  NPM-ALK in ALCL  ALK-amplified neuroblastoma  NPM-ALK ^F1174I, N1178H^ in Ba/F3 cells  VKORCIL1(e1)-ALK(e20)^T1151K^ lung cancer | [99,103,105-107] |
|  | Ceritinib^#^  (LDK378)  *ALK-inhibitor* | EML4-ALK ^G1202R^, and F1174V/C patient derived cells and Ba/F3 cells  NPM-ALK^G1128S, C1156F, I1171N/T, N1178H, E1210K, C1156F/D1230N^ in Ba/F3 cell model  ALK^L1152R, 1151Tins, G1202R, C1156Y, F1174C/L^  in Ba/F3 models, MET amplification, Neuregulin-1 overexpression | EML4-ALK ^L1196M, G1269A, I1171T and S1206Y^ in patient derived cells and Ba/F3 cells  NPM-ALK ^F1174I^ in Ba/F3 cells | [103,105,108,109] |
| *ALK* | NVP-TAE684  *ALK-inhibitor* | 1151T-ins  EML4(e13)-ALK(e20) in DFCI032 cell s and mice model due to co-activation of EGFR and ERBB2 | EML4-ALK, *EML4-*ALK^F1174L,^  *EML4-ALK*^L1196M-^amplified lung cancer  EML4 (e13)-ALK(e20) in H3122 and DFCI032 cells and mice model.  EML4(e6)-ALK(e20) H2228 cells and mice model) | [99,101,106,110,111] |
|  | Brigatinib^#^  (AP26113) *ALK&EGFR- inhibitor* | *EML4-ALK*^G1202R^, ^1151Tins^  NPM-ALK ^C1156F, I1171N/T, N1178H, E1210K, C1156F/D1230N^ in Ba/F3 cell model, Neuregulin-1 overexpression, ALK^G1202R^ | *EML4-ALK*^L1196M^  NSCLC xenograft model  NPM-ALK ^G1128S, F1174I^ in Ba/F3 cell model | [103,105,108,110] |
|  | ASP3026^#^  *ALK&ACK-inhibitor* | *EML4-ALK* in lung cancer  ALK 1151T-ins  NPM-ALK ^G1128S, C1156F,^ ^I1171N/T,^ ^F1174I,^ ^N1178H, E1210K, C1156F/D1230N^ in Ba/F3 cell model | *EML4-ALK*^L1196M^  NSCLC xenograft model | [99,105,108] |
|  | Ensartinib^#^  (X-396)  *ALK-inhibitor* | HCC78 (SLC34A2-ROS) expressing lung cancer cells, PC-9 (EGFR exon 19 del) expressing lung cancer cells, HepG2 hepatocellular carcinoma cells | EML4(e13)-ALK(e20) I cell n H3122 lung cancer  EML4(e6a/b)-ALK(e20) in H2228 lung cancer cell lines  EML4(e13)-ALK(e20)/C1156Y in Ba/F3 cells  EML4(e13)-ALK(e20)/L1196M in Ba/F3 cells  ALK F1174L in SY5Y neuroblastoma cells | [104] |
| *ALK* | Entrectinib  (NMS-E628) *ALK&ROS&NRTK- inhibitor* |  | EML4(e21)-ALK(e20) in CRC derived cells  CAD(e35)-ALK (e20) expressing CRC | [112,113] |
|  | Tanespimycin  (17-AAG)  *HSP90-inhibitor* | Cells lack of EML4-ALK fusion | RANBP2-ALK ^F1174L^ in IMT,  *EML4-ALK*^L1196M,^  ^C1156Y,^ ^L1152R, G1202R,^ ^S1206Y,^ ^1151Tins^ in Ba/F3 cells  *EML4-ALK*^L1196M^-amplified or HER2-amplified lung cancer | [99,101,110] |
|  | Lorlatinib^#^  (PF-6463922)  *ALK&ROS1-inhibitor* | NPM-ALK^G1128S, C1156F, I1171N/T, F1174I, N1178H, E1210K, C1156F/D1230N^ in Ba/F3 cell model  ALK^L1198F, C1166Y^ | NPM-ALK^F1174I^ in Ba/F3 cell model | [105] |
|  | Anti-PD-1/PD-L1 antibody |  | EML4-ALK expressing DFCI076 (crizotinib resistant) and A549 (crizotinib sensitive) NSCLC cell lines | [114] |
| *FGFR* | Dovitinib^#^  *Multi-TK-inhibitor* | ¶FGFR2^N550H/K/S/T,^ ^E566G/A,^ ^K642N,^ ^I548V,^ ^M538I,^ ^V565I,^ ^L618M,^ ^K660E,^ ^M536I,^ ^K642 V651I^ in BaF3 cells | FGFR2^S252W,^ ^N550K^ in endometrial cancer | [115-118] |
|  | BGJ398^#^  *Pan-FGFR-inhibitor* | EZR-ROS1 expressing cells | FGFR1-2 amplified,  FGFR2^W290C, S320C, K660E/N^ in SCLC  FGFR3^R248C, S249C^ in SCLC  FGFR2^S252W,^ ^N550K^ in endometrial cancer  FGFR2(e19)-CCDC6(e2) cholangiocarcinoma patient derived PDX model  FGFR2(e19)-AHCYL1(e5) or FGFR2(e19)-BHCC1(e3) expressing NIH3T3 cells and NIH3T3 transplanted mice | [117,119-123] |
|  | Ganetespib  *HSP90-inhibitor* |  | FGFR3-TACC3 expressing RT112 bladder cancer cell lines | [118] |
|  | PD173074  *FGFR-inhibitor* | ¶FGFR2^N550K/H/S,^ ^V565I,^ ^M536I,, M538I, I548V, V565I, E566G, L618M, K660E^ in BaF3 cells  FGFR1^V561M^  FGFR3^K652E and S249C^  FGFR3^S249C^fusion negative HT-1197 and  FGFR3^K652E^ fusion negative J82 bladder cancer cells | FGFR3-TACC3 expressing Rat1A and glioma stem-like cells, glioma xenograft  FGFR2(e19)-AHCYL1(e5) or FGFR2(e19)-BHCC1(e3) expressing NIH3T3 cells and NIH3T3 transplanted mice  FGFR3(e18)-BAIAP2L1(e2) expressing SW780 bladder cancer cell lines and xenografts  FGFR3(e18)-TACC3(e10) expressing HNE1 nasopharengeal carcinoma cells | [14,15,17,116,119,124] |
| *FGFR* | Pazopanib  *Multi-TK-inhibitor* |  | FGFR3-TACC3 expressing RT4 urothelial carcinoma cell line and xenograft,  FGFR3(e18)-BAIAP2L1(e2) expressing SW780 bladder cancer cell lines and xenografts | [17] |
|  | BGJ398 + Olaparib | Dbx9-Raf1 expressing BRCA1-deficient breast tumor bearing mice (BGJ398 alone) | FGFR2-DNM3 with BRCA1 deficient triple negative breast tumor-bearing mice | [125] |
|  | BGJ393+BKM120 |  | FGFR2-TNS1 expressing BRCA1-WT breast tumor-bearing mice | [125] |
|  | CH5183284/Debio-1347^#^  *FGFRs-inhibitor* | EML4-ALK expressing NIH-3T3 cells | FGFR3-BAIAP2L1 expressing bladder cancer cell line SW780, Rat-2_F3-B cells and xenograft model | [126] |
| *NTRK* | Entrectinib  (NMS-P626)  *Pan-NTRK-inhibitor* | TPM3-NTRK1, TPM3-NTRK1^G595R^, TPM3-NTRK1^G667C^ expressing NIH-3T3 cells | AFAP-NTRK2, SQSM1-NTRK2 expressing Ba/F3 cells | [100,108,127] |
|  | Larotrectinib  *Pan-TK-inhibitor* | TPM3-NTRK1, TPM3-NTRK1^G595R^, TPM3-NTRK1^G667C^ expressing NIH-3T3 cell lines |  | [127] |
|  | Merestinib^#^ (LY2801653)  *Pan-TK-inhinbitor* | TPM3-NTRK1^G595R^ | TPM3-NTRK1 expressing KM-12 colorectal cancer cells, NIH-3T3 cells, and PDX model, TPM3-NTRK1^G667C^ expressing NIH-3T3 cells, ETV6-NTRK3 expressing PDX model | [127] |
| *BRAF* | Olaparib + trametinib |  | DBX9-RAF1 expressing BRCA1-deficient breast tumor bearing mice | [125] |
|  | Trametinib  *MEK-inhibitor* | Longer treatment KIAA1549-BRAF or FAM131B-BRAF or BRAF^V600E^ expressing mice model due to increasing pMEK.  GNAI1-BRAF, MACF1- BRAF, MKRN1-BRAF, FXR1-BRAF, and CLCN6-BRAF expressing NIH3T3 cells. These fusions expressed in pediatric low-grade glioma. | KIAA1549-BRAF or FAM131B-BRAF or BRAF^V600E^ expressing NIH3T3 cells | [128] |
|  | Trametinib + everolimus |  | KIAA1549-BRAF or FAM131B-BRAF expressing NIH3T3 cells and PDX model  GNAI1-BRAF, MACF1- BRAF, MKRN1-BRAF, FXR1-BRAF, and CLCN6-BRAF expressing NIH3T3 cells | [128] |
| *BRAF* | Dabrafenib **  *BRAF^V600E^-inhibitor* | AHCYL2-BRAF | FAM114A2-BRAF and ATG7-BRAF expressing Ba/F3 cells | [100,129] |
|  | PLXPB-3  *BRAF^V600E^-inhibitor* |  | KIAA1549-BRAF in PA | [129] |
|  | Trametinib  *MEK-inhibitor* | SEPT3(e10)-BRAF(e8) expressing cells  CTNNBL1(e11)-RAF1(e11) in MCF7 and T47D breast cancer cells | SND1(e10)-BRAF(e9) in pancreatic acinar cell carcinoma  ZKSCAN1(e6)-BRAF(e10) in Spitzoid metastatic melanoma  ARMC10(e4)-BRAF(e11)  AGK(e2)-BRAF(e8)(less sensitive)  in melanoma PDX model | [130-133] |
|  | Trametinib  + olaparib |  | DHX9-RAF1 expressing BRCA1 deficient triple negative breast tumor-bearing mice | [125] |
| *RET* | Cabozantinib | RET V804M | NCOA4(e8)-RET(e12) expressing NIH3T3 cells and PDX model in ER+/PR-/HER2+breast cancer | [134] |
|  | Vandetanib^#^  *Multi-TK-inhibitor* | *RET*^V804M/L^ | CCDC6(e1)-RET(e12) in lung adenocarcinoma LC-2/ad cells | [135] |
|  | Ponatinib (AP24534)  *Multi-TK-inhibitor* | CCDC6-RET fusion expressing and NRAS Q61K mutant PR1 cells derived from LC-2/ad cells,  CCDC6-RET fusion expressing and wild-type EGFR and AXL activating PR2 cells derived from LC-2/ad | CCDC6-RET fusion expressing LC-2/ad lung adenocarcinoma cells | [136] |
| *MET* | Crizotinib  *MET-inhibitor* |  | BAIAP2L1-MET and  TFG-MET expressing Ba/F3 cells | [100] |
| *AKT* | MK-2206  *AKT-inhibitor* | S6KC1(e6)-AKT(e3) in HR+ breast cancer cells (MCF7 and T47D) |  | [133] |

*Non-mutant selective inhibitor; **mutant selective inhibitor; ^#^ ClinicalTrial.gov; ^Clinical trial in Japan; There are much more selective or panTKIs are under development.
